# Metabolic alterations in alga *Chlamydomonas reinhardtii* exposed to nTiO_2_ materials†

**DOI:** 10.1039/d2en00260d

**Published:** 2022-07-01

**Authors:** Wei Liu, Mengting Li, Weiwei Li, Arturo A. Keller, Vera I. Slaveykova

**Affiliations:** University of Geneva, Faculty of Sciences, Earth and Environment Sciences, Department F.-A. Forel for Environmental and Aquatic Sciences, Environmental Biogeochemistry and Ecotoxicology Uni Carl Vogt, 66 Blvd Carl-Vogt CH 1211 Geneva Switzerland vera.slaveykova@unige.ch; Bren School of Environmental Science & Management, University of California Santa Barbara California 93106-5131 USA

## Abstract

Nano-sized titanium dioxide (nTiO_2_) is one of the most commonly used materials, however the knowledge about the molecular basis for metabolic and physiological changes in phytoplankton is yet to be explored. In the present study we use a combination of targeted metabolomics, transcriptomics and physiological response studies to decipher the metabolic perturbation in green alga *Chlamydomonas reinhardtii* exposed for 72 h to increasing concentrations (2, 20, 100 and 200 mg L^−1^) of nTiO_2_ with primary sizes of 5, 15 and 20 nm. Results show that the exposure to all three nTiO_2_ materials induced perturbation of the metabolism of amino acids, nucleotides, fatty acids, tricarboxylic acids, antioxidants but not in the photosynthesis. The alterations of the most responsive metabolites were concentration and primary size-dependent despite the significant formation of micrometer-size aggregates and their sedimentation. The metabolic perturbations corroborate the observed physiological responses and transcriptomic results and confirmed the importance of oxidative stress as a major toxicity mechanism for nTiO_2_. Transcriptomics revealed also an important influence of nTiO_2_ treatments on the transport, adenosine triphosphate binding cassette transporters, and metal transporters, suggesting a perturbation in a global nutrition of the microalgal cell, which was most pronounced for exposure to 5 nm nTiO_2_. The present study provides for the first-time evidence for the main metabolic perturbations in green alga *C. reinhardtii* exposed to nTiO_2_ and helps to improve biological understanding of the molecular basis of these perturbations.

## Abstract 2

Combination of transcriptomics, metabolomics and physiology studies highlighted the nanoparticle size- and concentration-dependent disturbance in algal metabolism induced by nTiO_2_.

## Graphical abstract

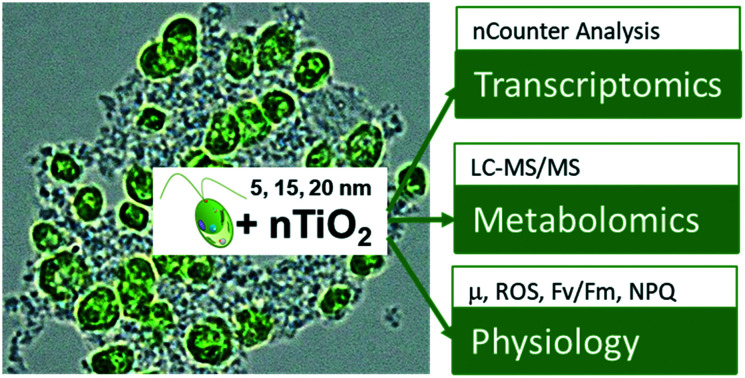

**Environmental significance**

A significant knowledge gap exists regarding the metabolic perturbations induced by engineered nanomaterials on phytoplankton species, in spite of their essential role in global biogeochemical cycles and food-web dynamics in aquatic environments. We have combined targeted metabolomics, transcriptomics and physiological response studies to explore quantitatively for the first time the metabolic alterations in green algae exposed to increasing concentrations of nano-sized titanium dioxide (nTiO_2_) materials with three different primary sizes. We selected nTiO_2_ as one of the most commonly used nanomaterials. Results revealed that exposure to nTiO_2_ at concentrations higher than those usually found in freshwater environments induces significant alteration of the metabolism of amino acids, nucleotides, fatty acids, tricarboxylic acids and antioxidants, as well as disturbance of the global nutrient status of the algae. For most of the responsive metabolites the changes in the abundance upon nTiO_2_ treatments were exposure concentration and primary size dependent.

## Introduction

Nano-sized titanium dioxide (nTiO_2_) is one of the top five most commonly used materials.^1,2^ Recent measurements revealed that the concentrations of Ti-containing nanoparticles range between 10^4^ and 10^7^ nanoparticles per mL (corresponding to mass concentrations <10 μg L^−1^) in global surface waters.^3^ However in some hotspots, such as the runoff from the façade of a new building, much higher concentrations were detected.^4^ Once released into the environment, nTiO_2_ are subject of different physical, chemical and biological transformations, depending on the nanoparticle characteristics, environmental and biological factors.^5–7^ nTiO_2_ are considered as moderately stable in freshwater (global stability index of 0.46 for artificial freshwater).^8^ Accompanying the well-established use and increasing environmental concentrations of nTiO_2_, a significant amount of research has been conducted to assess their potential toxicity to different biota, including phytoplankton species.^9–16^ nTiO_2_ have been classified as “harmful” for the environment.^17^ Evidence is accumulating that nTiO_2_ can induce the enhanced production of reactive oxygen species (ROS) and oxidative stress,^7,9,18–21^ the disruption of the cell membrane and interruption of energy transductions,^22^ DNA and genotoxic damage^23,24^ and the reduction of light available to algal cells due to the trapping of the algal cells in the nTiO_2_ aggregates.^25^ For a given organism, the toxicity of nTiO_2_ depends on the size, crystalline structure and surface chemistry of nanoparticles in water.^26–29^ The advances of ‘omic’ technologies allow a deeper insight into the metabolic perturbation in different phytoplankton species and the modes-of-action of environmental contaminants, including metal-containing nanoparticles.^30–35^ For instance, nCeO_2_ with different surface coatings affected the transcripts encoding flagella, indicating an effect on algal mobility.^36^ Exposure to MoS_2_ nanosheets altered significantly the nitrogen metabolism in cyanobacterium *Nostoc*.^37^ Similarly, exposure of golden-brown alga *Poterioochromonas malhamensis* to nAg and released silver ions induced perturbation of the metabolism of amino acids, nucleotides, fatty acids, tricarboxylic acids, photosynthesis and photorespiration.^38^ Dissolvable nAg decreased the abundance of d-galactose, sucrose, and d-fructose, carbohydrates involved in the synthesis and repair of cell walls, in a concentration dependent manner in *Scenedesmus obliquus*.^39^ nAg affected the phosphorus assimilation metabolism in green alga *Chlorella vulgaris*^40^ as well as altered the arginine and proline metabolism, indole alkaloid biosynthesis, and phospholipid metabolism in cyanobacterium *Microcystis aeruginosa*.^41^ nAg exposure increased the levels of transcripts encoding components of the cell wall and the flagella *C. reinhardtii*.^42^ Despite these advancements, little published research has focused on the cellular and molecular mechanisms involved in the stress tolerance of microalgae exposed to nTiO_2_ with different primary sizes. The only “omic” study revealed that nTiO_2_ raised the levels of transcripts encoding subunits of the proteasome, suggesting proteasome inhibition in *C. reinhardtii*.^42^

The main objective of the present study is to obtain a novel comprehensive understanding about the metabolic perturbations induced in microalgae exposed to nTiO_2_. To this end, green alga *C. reinhardtii*, a representative aquatic primary producer, was exposed to increasing concentrations (2 to 200 mg L^−1^) of nTiO_2_ with primary sizes of 5, 15 and 20 nm for 72 h. We hypothesized that the observed metabolic perturbations will be dependent on the primary size and exposure concentration of nTiO_2_. To verify this hypothesis, we have performed a targeted metabolomics study. The metabolomics results were further corroborated with the biological responses at physiological (growth inhibition, oxidative stress, membrane damage and changes in photosynthetic activity) and transcriptomic levels. In addition, the behaviour of nTiO_2_ in the exposure medium was characterized in terms of aggregation and sedimentation. The findings of this exploratory study contribute to the improvement of the current knowledge regarding nTiO_2_ toxicity pathways in this species, and possibly other species of microalgae.

## Experimental

### nTiO_2_ materials

Powdered nano-sized TiO_2_ particles with different primary sizes (anatase 5 nm (A5), anatase 15 nm (A15) and anatase/rutile 20 nm (AR20)) were purchased from Nanostructured & Amorphous Materials Inc, USA. The primary characteristics of the materials as provided by the manufacturer are given in Table S1, ESI.† Stock suspensions of 2.0 g L^−1^ nTiO_2_ were prepared by dispersing nanoparticles in ultrapure water (Millipore, Darmstadt, Germany) using sonication for 10 min (50 W L^−1^ at 40 kHz). The stock suspensions were used to prepare suspensions of nTiO_2_ in the exposure medium containing 2, 20, 100 and 200 mg L^−1^ of A5, A15 or AR20. The exposure medium consisted of 8.2 × 10^−4^ M CaCl_2_·2H_2_O, 3.6 × 10^−4^ M MgSO_4_·7H_2_O, 2.8 × 10^−4^ M NaHCO_3_, 1.0 × 10^−4^ M KH_2_PO_4_ and 5.0 × 10^−6^ M NH_4_NO_3_ with a pH of 7.0 ± 0.2 and an ionic strength of 2.75 mM, as in our previous studies.^21,43^ The selected test concentrations of nTiO_2_ are much higher than those found in freshwater environments,^3^ but cover a range of ecotoxicologically relevant concentrations.^44^

### Characterization of nTiO_2_ in algal exposure medium

The *Z*-average hydrodynamic diameter and zeta potential of the three types of nTiO_2_ materials suspended in the exposure medium were measured at 72 h using a Malvern Zetasizer Nano-ZS (Malvern Instruments, Worcestershire, UK) at 20 °C. Results are the means of 3 sample measurements, 5 runs for each. The suspended fraction of the nTiO_2_ suspensions was determined by using a UV-vis spectrophotometer (PerkinElmer UV/visible spectrophotometer Lambda 365, wave range of 200–800 nm) at the beginning of the experiment and at 72 h. The suspended fraction was determined as the ratio between the final (*A*) and initial absorbance (*A*_0_) at the maximum absorption peak wavelength of 243 nm (*A*/*A*_0_) as detailed previously.^43^

### Bioassays with *Chlamydomonas reinhardtii*

Wild-type *C. reinhardtii* (CPCC11, Canadian Phycological Culture Centre, Waterloo, Canada) was grown axenically at 20.2 ± 0.5 °C, 115 rpm and 110 μmol phot m^−2^ s^−1^. Algal cells were cultured in 4× diluted tris-acetate-phosphate (4× TAP) medium^45^ until mid-exponential growth (62 hours post-inoculation), centrifuged (10 minutes, 1300*g*), rinsed and re-suspended (∼10^6^ cells per mL) in the exposure medium enriched with 2, 20, 100, and 200 mg L^−1^ A5, A15 or AR20 for 72 h. Negative controls (NC) in the absence of nTiO_2_ were also performed. All the experiments were performed in 3 independent biological replicates. Changes in algal growth and physiology (excessive ROS generation, membrane damage and alteration of photosynthetic activity), alteration of key metabolites induced by nTiO_2_ exposure and transcriptomic response were determined as presented below.

### Evaluation of the nTiO_2_ effect on algal growth and physiology

The effect of nTiO_2_ on the algal growth was assessed following the changes of the algal cell densities by flow cytometry (FCM). The measurements were performed with a BD Accuri C6 flow cytometer equipped with a CSampler (BD Biosciences, San Jose, CA). The 488 nm argon excitation laser and fluorescence detection channels with band pass emission filters at 530 ± 15 nm (FL1), 585 ± 20 nm (FL2) and a long pass emission filter for >670 nm (FL3) were used. Data acquisition and analysis were performed with the BD Accuri C6 Software 264.15. The primary threshold was set to 20 000 events on FSC-H. Information on algal cell densities and chlorophyll fluorescence (in FL3) was obtained in a single run after 72 h incubation. Algal cells were discriminated from nTiO_2_ aggregates with similar size by applying a gating strategy, as described in our earlier study.^21^ The ROS generation and membrane damage were examined using the fluorescent probes CellROX® green (Life Technologies Europe B.V., Zug, Switzerland) and propidium iodide (PI) (Sigma-Aldrich, Buchs, Switzerland) and the number of affected cells was followed by FCM. The detailed staining procedures and gating strategies are presented in our previous studies.^21,46^ The photosynthetic activity of *C. reinhardtii* was determined prior to and after nTiO_2_ treatment using a Multiple Excitation Wavelength Chlorophyll Fluorescence Analyzer (Multi-Color-PAM, Walz, Germany). The maximal fluorescence yield of photosystem II, Fm, and maximal variable fluorescence, Fv (Fv/Fm) and non-photochemical quenching (NPQ) were measured after 72 h exposure following a 20 min dark acclimation. These parameters are well-known indicators for alteration of photosynthetic activity by different biotic and abiotic stressors.^38^

### Assessment of the metabolic response by liquid chromatography-mass spectrometry targeted metabolomics

Changes in concentrations of key primary metabolites in *C. reinhardtii* exposed to the three nTiO_2_ materials of different primary sizes at four concentrations (2, 20, 100, and 200 mg L^−1^) and unexposed controls were determined by liquid chromatography-mass spectrometry (LC-MS) targeted metabolomics. At the end of the exposure for 72 h, the cells were placed in liquid nitrogen to stop metabolic activity, then frozen at −80 °C for 24 h and freeze-dried. Eighty metabolites, including antioxidants, amino acids, organic acids/phenolics, nucleobase/side/tide, sugar/sugar alcohols and fatty acids, were extracted in 80% methanol containing 2% formic acid, following a previously developed methodology.^38,47^ Targeted analyses of these metabolites were performed using an Agilent 6470 liquid chromatography triple quadrupole mass spectrometer according to previously established methods.^47–50^ The absolute concentrations of the metabolites in each sample were quantified using 7 point calibration standards, with isotopically-labeled metabolites as internal standards.

Statistical analyses of the metabolomics data were performed for exposed algae and control using MetaboAnalyst 5.0.^51^ One-way analysis of variance (ANOVA) followed by Fisher's LSD *post hoc* analysis with *p* < 0.05 was completed to screen for metabolites differing between nTiO_2_ treatments and controls, as well as differing between different nTiO_2_ concentrations or primary sizes. Unsupervised principal component analysis (PCA) and supervised partial least squares-discriminant analysis (PLS-DA) were performed to get a global overview of the metabolic changes obtained in treatments with nTiO_2_ of different primary sizes and different concentrations. Metabolites with a variable importance in the projection (VIP) greater than 1 were regarded as significant and responsible for group separation.^52^ Metabolites significantly dysregulated by exposure to nTiO_2_, as identified *via* ANOVA and PLS-DA, were further considered as responsive metabolites. Pathway analyses were performed with MetaboAnalyst 5.0 with respect to the KEGG pathway built-in metabolic library of green alga *Chlorella variables.*^53^ Pathways with threshold >0.1 were considered as significantly dysregulated.^54,55^

### Assessment of transcriptomic response using nCounter

To corroborate the results of targeted metabolomics, 117 transcripts were selected for analysis, including those involved in the response and toxicity pathway of *C. reinhardtii* to different pollutants^56^ available in the Gene Expression Omnibus database (GSE65109). 94 of the transcripts have an annotation in MapMan ontology,^57^ representing amino acid metabolism (16 transcripts), carbohydrate metabolism (17), stress (16), transport (30), metal binding (5) and photosynthesis (9). All selected transcripts were regulated by more than 2-fold in at least three conditions, and all of them had annotations to genes with known functions. Probes were designed and synthesized by NanoString nCounter Technologies (Table S2†). This technology offers a medium-throughput quantitative approach to study differential transcript expression. Total RNA was extracted from the *C. reinhardtii* exposed to the three types of nTiO_2_ materials at two concentrations (2 and 20 mg L^−1^) for 72 h. These concentrations were chosen based on the preliminary tests at exposure concentrations of 2, 20, 100 and 200 mg L^−1^ of nTiO_2_ and considering the quality of extracted RNA. Untreated cells were used as control. For each replicate, approximately 100 mg fresh weight of algal cells from the control and experimental groups was harvested by centrifugation at 4 °C at 3200 × *g* for 5 min. Total RNA was extracted following the previously established protocol for *C. reinhardtii*.^58,59^ The concentration of RNA was determined using the Qubit RNA Broad Range Assay Kit and a Qubit 2.0 fluorometer (Thermo Fisher Scientific, USA) following the manufacturer's instructions. The purity of RNA samples was estimated by assaying 2 μL of total RNA extract on a NanoDrop 2000c spectrophotometer (Thermo Scientific, USA). The quality of the RNA was confirmed (A 260/A 280: 1.8–2.0; A 260/A 230: 1.8–2.2).

100 ng of total RNA was hybridized with multiplexed Nanostring probes and samples were processed according to a published procedure.^60^ Background correction was made by subtracting from the raw counts the mean ± 2 standard deviation of counts obtained with negative controls. Values <1 were fixed to 1 to avoid negative values after log transformation. Then, counts for target transcripts were normalized with the geometric mean of six housekeeping genes (Cre06_g260950_t1_2.1, Cre06_g272950_t1_1.1, Cre08_g370550_t1_1.1, Cre09_g411100_t1_2.1, Cre12_g519180_t1_1, Cre06_g260950_t1_2.1) selected as the most stable using the geNorm algorithm.^61^ Normalized data were log 2 transformed for further analyses.

Statistical analysis of nCounter data was carried out with R, a free software environment available at https://www.r-project.org. After importing the normalized data, the significance of differential transcript expression between the groups was determined by computing the moderated *t*-statistics and false discovery rate (FDR) with the BioConductor package limma, available at https://www.bioconductor.org. *p*-Values were corrected for multiple testing by the use of the FDR method.^62^ A significance threshold *p* < 0.05 associated with a fold change value of 2 or more was applied. Graphical representations were computed in GraphPad Prism 9.0 (Prism, GraphPad Software, San Diego).

## Results and discussion

### Characterization of nTiO_2_ suspensions in the exposure medium

**Fig. 1:**
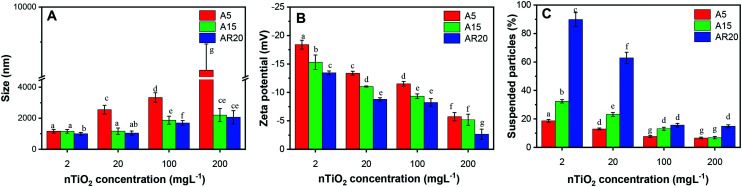
Characterization of the stability at 72 h in the exposure medium of nTiO_2_ at concentrations of 2, 20, 100 and 200 mg L^−1^. (A) TiO_2_*z*-average hydrodynamic diameter; (B) TiO_2_ zeta potential; (C) sedimentation of nTiO_2_. Different letters indicate statistically significant differences between the values as obtained by ANOVA and a Tukey's *post hoc* test (*p* < 0.05).

Since the stability of nTiO_2_ dispersions in exposure medium plays an important role in the induced biological responses, we characterized nTiO_2_ suspensions in the exposure medium in terms of their aggregation and sedimentation (Fig. 1). All three types of nTiO_2_ aggregated substantially in the algal exposure medium at 72 h even at 2 mg L^−1^, forming aggregates with sizes above 1000 nm (Fig. 1A). An increase of nTiO_2_ concentration resulted in a rise in aggregate size, which was particularly pronounced for the A5 material with a primary size of 5 nm. The changes in average size with increasing concentration were less pronounced for A15 and AR20. A5 formed much bigger aggregates than A15 and AR20 at higher concentrations. For example, the *z*-average hydrodynamic diameter of A5 was about 3.9 times higher than those of A15 and AR20 in suspensions containing 200 mg L^−1^. No significant difference was found between the aggregate size of A15 and AR20 at the four tested concentrations. All nTiO_2_ suspensions in exposure medium exhibited a negative zeta potential value (Fig. 1B): −18.1 mV for A5, −15.3 mV for A15 and −13.4 mV for AR20 at a concentration of 2 mg L^−1^. Increasing nTiO_2_ concentration resulted in a significant decrease in the absolute values of the zeta potential for the three tested nTiO_2_ materials, which was consistent with the formation of bigger nTiO_2_ aggregates. As shown in our preceding studies, the three nTiO_2_ materials form rapidly micrometer aggregates in the exposure medium (2 h exposure,^21^) and the size of the aggregates stayed stable over the time interval 24–96 h.^43^

The percentage of suspended TiO_2_ decreased with increasing concentration (Fig. 1C), reflecting the larger aggregates formed at higher concentrations and their sedimentation rates. For example, only about 19%, 32% and 88% of particles for A5, A15 and AR20, respectively, were still suspended after 72 h at a concentration of 2 mg L^−1^. Our previous kinetic study revealed that the micrometer aggregates of nTiO_2_ settled down rapidly within the first 24 h and the percentage of the suspended particles tended to stay constant from 24 to 96 h.^43^ The above observations are consistent with the fact that the nTiO_2_ suspensions at higher concentrations are more susceptible to aggregation due to the increased collision probability between the particles.^63^ It is currently accepted that the aggregation state of nTiO_2_ suspensions is a result of the van der Waals attraction forces,^64^ Coulomb repulsion caused by the surface charge of the particles or the action of the electrostatic double layer, and steric hindrance.^65^

### Effect of nTiO_2_ treatments on algal physiology

**Fig. 2:**
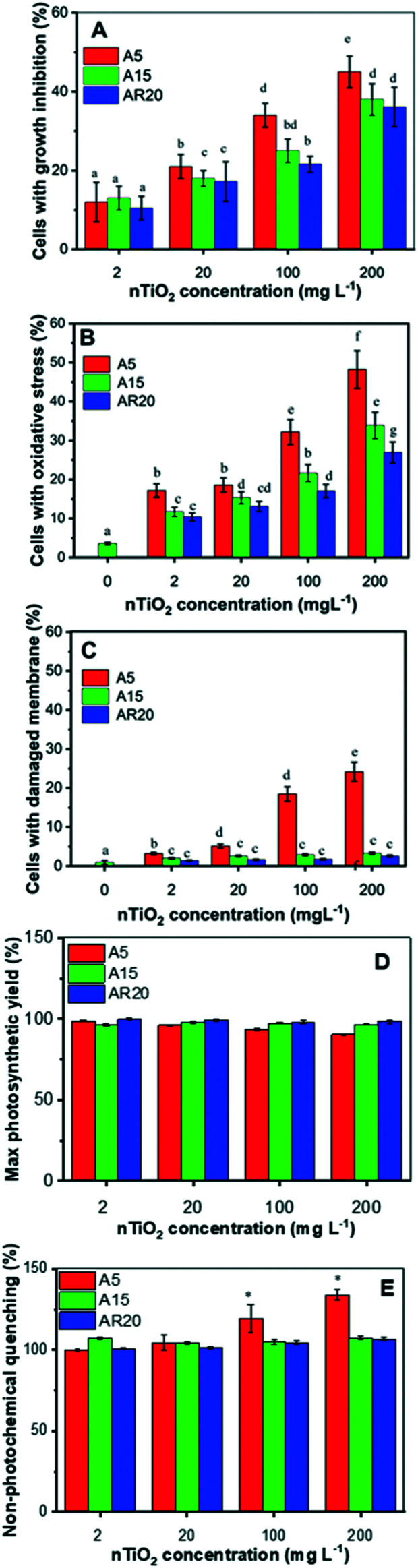
Effect of nTiO_2_ on (A) the percentage of cells with inhibited growth, (B) the percentage of cells with oxidative stress, (C) the percentage of cells with membrane damage, (D) cells with the maximum quantum yield of photosystem II (Fv/Fm) as a % of control, and (E) non-photochemical quenching (NPQ) as a % of control in *C. reinhardtii* exposed to three different materials: A5, A15 and AR20. (A and B) Different letters indicate statistically significant differences between the values as obtained by ANOVA and a Tukey's *post hoc* test (*p* < 0.05). (C and D) * indicate statistically significant differences between the values as obtained by ANOVA and a Tukey's *post hoc* test (*p* < 0.05).

All three tested nTiO_2_ materials induced growth inhibition in less than 50% of the exposed alga and the effect was concentration dependent (Fig. 2A). Exposure to A5 resulted in the most important growth inhibition, compared with the A15 and AR20 treatments, showing that the nTiO_2_ with lower size inhibited the growth of *C. reinhardtii* more significantly (*p* < 0.05). These observations agree with the finding that the toxicity outcome of exposure to nTiO_2_ depends on the primary size.^20,27,29^ These findings would also suggest that *C. reinhardtii* is more tolerant to nTiO_2_ in comparison with other green algae and diatoms.^20,28,29,44^ However, direct comparison is not possible given different exposure medium compositions and different nTiO_2_ materials used. The generation of excessive cellular ROS concentrations is considered the most likely toxicity mechanism of nTiO_2_.^20^ Here all three types of nTiO_2_ induced oxidative stress in *C. reinhardtii* even at the lowest tested concentration of 2 mg L^−1^ (Fig. 2B). The oxidative stress became more pronounced with increasing nTiO_2_ concentration for all tested materials. A5 induced stronger oxidative stress than A15 and AR20, as already observed at short term exposure.^21^

Changes in membrane permeability induced by 72 h-exposure to A15 and AR20 were negligible. The percentage of affected cells was comparable to unexposed controls. However, exposure of *C. reinhardtii* to A5 resulted in a significant percentage of cells with membrane damage at a nTiO_2_ concentration of 200 mg L^−1^ (Fig. 2C). This result is also consistent with the observed trapping of the algal cells in aggregates of A5 at 96 h exposure.^43^

nTiO_2_ treatments had no measurable effect on algal photosynthesis. No significant difference (*p* > 0.05) of the Fv/Fm and NPQ values was found in all treatments except for A5 at a concentration of 200 mg L^−1^ in comparison with the control after 72 h (Fig. 2D and E). The decrease in Fv/Fm reveals an inhibition in algal photosynthetic activity related to light energy utilization caused by 200 mg L^−1^ A5. In agreement with Fv/Fm, no significant differences in NPQ were observed after exposure to the three nTiO_2_ materials. A significant effect (*p* < 0.05) on NPQ was found only upon exposure to 200 mg L^−1^ A5, indicating that the absorbed light energy exceeded the capacity of its utilization in photosynthetic processes, considered as a primary defense mechanism employed to dissipate the stress.

### Overview of metabolic profiles in *C. reinhardtii* under different nTiO_2_ treatments

**Fig. 3:**
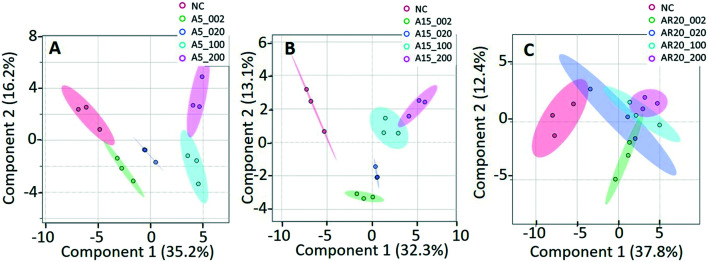
Analysis of metabolic response of *C. reinhardtii* treated with increasing concentrations of three nTiO_2_ materials by partial least-squares discriminate analysis (PLS-DA) score plots: treatments with: (A) 5 nm nTiO_2_ 2 mg L^−1^ (A5_2), 20 mg L^−1^ (A5_20), 100 mg L^−1^ (A5_100) and 200 mg L^−1^ (A5_200); (B) 15 nm nTiO_2_ 2 mg L^−1^ (A15_2), 20 mg L^−1^ (A15_20), 100 mg L^−1^ (A15_100) and 200 mg L^−1^ (A15_200); (C) 20 nm nTiO_2_ (AR20): 2 mg L^−1^ (AR20_2), 20 mg L^−1^ (AR20_20), 100 mg L^−1^ (AR20_100) and 200 mg L^−1^ (AR20_200); negative control (NC). Data were row-wise normalized using probabilistic quotient normalization by reference groups, non-transformed and autoscaled. The score plots and heatmap were generated using MetaboAnalyst 5.0 (https://www.metaboanalyst.ca/).

Of a total of 80 metabolites analyzed, 50 were found above their limits of detection and determined in the controls and treatments with nTiO_2_ of different primary sizes and concentrations. A general overview of the different treatment groups was obtained by unsupervised PCA (Fig. S1†) and supervised PLS-DA for concentration treatments (Fig. 3) and different size treatments (Fig. S2† and 4). The PLS-DA score plot (Fig. 3A and B) showed excellent separation of A5 and A15 treatments and unexposed controls, as well as treatments with different nTiO_2_ concentrations, highlighting the importance of tracking the alteration of the metabolic response with exposure concentrations. For the AR20 exposure, the separation of the nTiO_2_ treatments from controls and among the treatments was not very good (Fig. 3C). Based on a VIP score >1 (Fig. S3†) and ANOVA (Tables S3–S5†), a total of 26 responsive metabolites were identified to be significant in the A5-treatments in comparison with the untreated control; 25 for A15 and 26 for AR20.

**Fig. 4:**
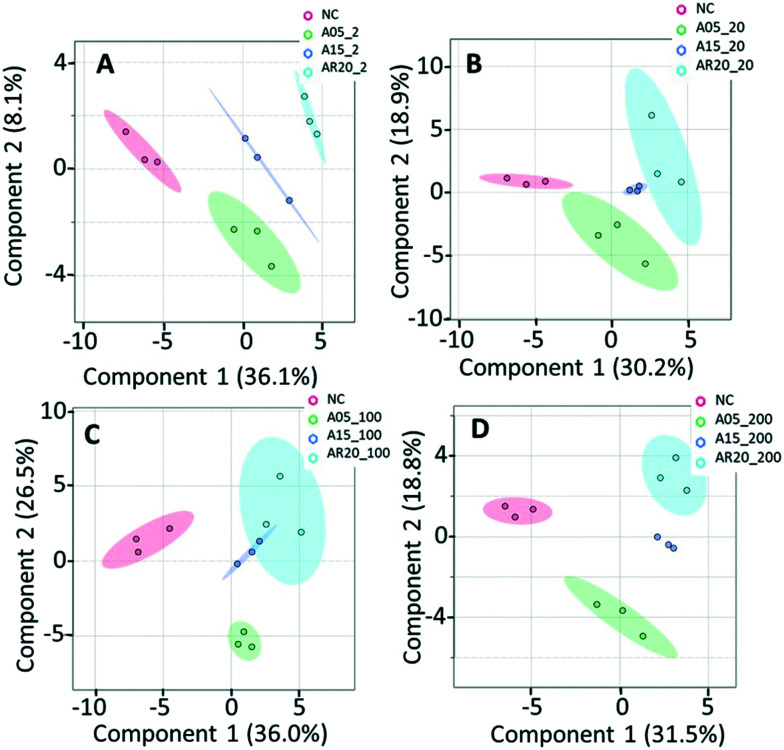
PLS-DA of metabolic response of *C. reinhardtii* treated with nTiO_2_ materials of different primary sizes. Treatments with: (A) 2 mg L^−1^ of nTiO_2_ with a size of 5 nm (A5_002), 15 nm (A15_002) and 20 nm (AR20_002); (B) 20 mg L^−1^ of nTiO_2_ with a size of 5 nm (A5_020), 15 nm (A15_020) and 20 nm (AR20_020); (C) 100 mg L^−1^ of nTiO_2_ with a size of 5 nm (A5_100), 15 nm (A15_100) and 20 nm (AR20_100); (D) 200 mg L^−1^ of nTiO_2_ with a size of 5 nm (A5_200), 15 nm (A15_200) and 20 nm (AR20_200); negative control (NC). Data were row-wise normalized using probabilistic quotient normalization by reference groups, non-transformed and autoscaled. The score plots are generated by MetaboAnalyst 5.0 (https://www.metaboanalyst.ca/).

The results of the supervised PLS-DA for treatments with a given concentration of nTiO_2_ of different primary sizes (Fig. 4) showed very good separation between the control and different primary size treatments for all concentrations, demonstrating the importance of considering the primary size of nTiO_2_ in metabolic perturbations. Based on a VIP score >1 (Fig. S4†) and ANOVA (Tables S6–S9†), a total of 28 responsive metabolites were found for 2 mg L^−1^, 21 for 20 mg L^−1^, 34 for 100 mg L^−1^ and 29 for 200 mg L^−1^ nTiO_2_, for different size treatments. With a small exception, the abundance of the same metabolites was affected in different treatments with a rather clear size dependence for the same initial nTiO_2_ concentration. It is necessary to keep in mind that in addition to the differences in size there is a small difference in the crystalline structure: A5 and A15 are composed of anatase, whereas AR20 contains 80–90% anatase and 10–20% rutile. nTiO_2_ in anatase crystal structure was shown to be more toxic for algae than rutile.^28^

Overall, most of the responsive metabolites were common for different nTiO_2_ concentration- and size-treatments. They included mainly amino acids, antioxidants, some fatty and carboxylic acids and nucleobases/sides/tides. However, the intensity of the responses and size- or concentration-dependences differed, as discussed further.

The responsive metabolites were also grouped by heatmap clustering. In different concentration treatments (Fig. S5–S7†), three large groups were identified corresponding to (i) metabolites with *higher abundance* in algae treated with nTiO_2_ than in the control (12 for A5, 12 for A15 and 15 for AR20). In the group, the metabolites with increasing abundance with the nTiO_2_ concentrations were 9 for A5, 5 for A15, and 5 for AR20; (ii) metabolites with *lower abundance* in nTiO_2_ treatments than in the untreated control (11 for A5, 11 for A15 and 12 for AR20). Within this group, the number of metabolites with decreasing abundance with nTiO_2_ concentrations was 9 for A5, 11 for A15, and 10 for AR20; and (iii) metabolites with no clear concentration dependence.

Clustering of the treatments as a function of the primary size of nTiO_2_ revealed five clusters including (Fig. S8–S11†): (i) metabolites with *higher abundance* than the control; increase of metabolite concentrations with primary size (metabolites with the *highest abundance* in AR20 treatments: 11 at 2 mg L^−1^, 8 at 20 mg L^−1^, 3 at 100 mg L^−1^, 1 at 200 mg L^−1^); (ii) metabolites with *higher abundance* than the control, decrease of metabolite concentrations with primary size (metabolites with the *highest abundance* in A5 treatments: 2 at 2 mg L^−1^, 2 at 20 mg L^−1^, 11 at 100 mg L^−1^, 8 at 200 mg L^−1^); (iii) metabolites with *lower abundance* than the control; decrease of the metabolite concentrations with the primary size (metabolites with the *lowest abundance* in AR20 treatments: 7 at 2 mg L^−1^, 7 at 20 mg L^−1^, 7 at 100 mg L^−1^, 6 at 200 mg L^−1^); (iv) metabolites with *lower abundance* than the control; increase of the metabolite concentration with primary size (metabolites with the lowest abundance in A5 treatments – 6 at 2 mg L^−1^, 3 at 20 mg L^−1^, 6 at 100 mg L^−1^, 5 at 200 mg L^−1^). The number of metabolites with the highest or lowest abundance at 2 and 20 mg L^−1^ for AR20 treatments could be related to the much higher percentage of AR20 dispersed in the suspensions of 2 and 20 mg L^−1^ nTiO_2_ than 100 and 200 mg L^−1^ nTiO_2_ treatments; (v) metabolites with no clear concentration dependence.

Furthermore, the responsive metabolites identified by PLS-DA and ANOVA corresponded to 11impacted pathways (Fig. S12 and S13†). Interestingly, the number of the significantly impacted pathways increased with the decrease of the primary size (Fig. S12†) and the increase of nTiO_2_ exposure concentrations (Fig. S13†). The glutathione metabolism, alanine, aspartate and glutamate metabolism, cysteine and methionine metabolism, phenylalanine metabolism and arginine biosynthesis were commonly affected in different treatments. Exposure to A5 resulted in the strongest impact on the glutathione metabolism.

### Overview of transcriptomic profiles in *C. reinhardtii* under different nTiO_2_ treatments

**Fig. 5:**
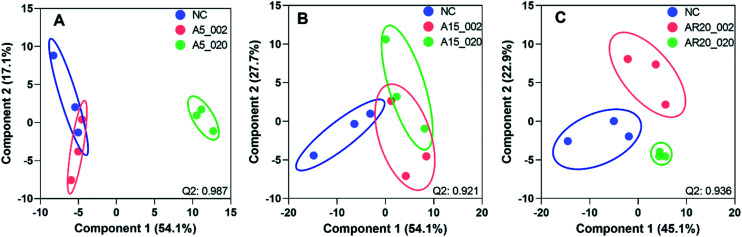
PLS-DA of transcript expression of *C. reinhardtii* exposed to 2 and 20 mg L^−1^ of three types of nTiO_2_. (A) Treatments with A5 at 2 mg L^−1^ (A5_002) and 20 mg L^−1^ (A5_020). (B) Treatments with A15 at 2 mg L^−1^ (A5_002) and 20 mg L^−1^ (A5_020). (C) Treatments with AR20 at 2 mg L^−1^ (A5_002) and 20 mg L^−1^ (A5_020); negative control (NC). The significance of differential transcript expression between the groups was determined by computing the moderated *t*-statistics and FDR with the BioConductor package limma in R. Q2 values for cross validation of the models are also given.

PCA and PLS-DA of the differences in transcript expression between the unexposed controls, treatments with nTiO_2_ of different concentrations (Fig. S14† and 5) and different primary sizes (Fig. S15† and 6) were conducted. A good separation of A5 and AR20 treatments from the control as well as treatments with different concentrations of nTiO_2_ was observed (Fig. 5A and C). Poor separation between A15 treatments and the control was found (Fig. 5B).

**Fig. 6:**
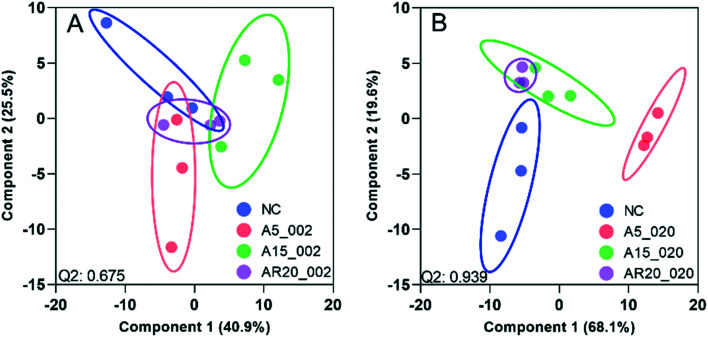
PLS-DA of transcript expression of *C. reinhardtii* exposed to nTiO_2_ of 5, 15 and 20 nm primary size at 2 and 20 mg L^−1^. (A) Treatments with A5 at 2 mg L^−1^ of A5 (A5_002), A15 (A 15_002) and AR20 (AR20_002). (B) Treatments with A5 at 20 mg L^−1^ of A5 (A5_020), A15 (A 15_020) and AR20 (AR20_020); negative control (NC). The significance of differential transcript expression between the groups was determined by computing the moderated *t*-statistics and FDR with the BioConductor package limma in R. Q2 values for cross validation of the models are also given.

At 2 mg L^−1^ no separation between the treatments with different sizes and controls was observed (Fig. 6A). However, at 20 mg L^−1^, good separation of the treatments with all three nTiO_2_ materials and controls was found. In addition, the distribution of principal components of A5 was clearly separate from A15 and AR20 (Fig. 6B). These results highlighted that the three nTiO_2_ altered significantly the transcriptomic expression in *C. reinhardtii* in a primary size dependent way at concentrations of 20 mg L^−1^.

Of the 117 transcripts tested with nCounter, only a few transcripts allowed discrimination between the treatments. For A5 treatment, the abundance of 3 and 49 transcripts was significantly altered upon exposure to A5 at 2 (Fig. S16A†) and 20 mg L^−1^ (Fig. S16B†), respectively. However, the expression of few transcripts was significantly altered in A15 and AR20 treatments: 9 for A15 at 2 and 20 mg L^−1^ (Fig. S16C and D†), 3 and 8 for AR20 at 2 and 20 mg L^−1^ (Fig. S16E and F†), respectively. This analysis confirmed that for exposure to 20 mg L^−1^, nTiO_2_ of smaller primary size (A5) has a significantly larger effect on transcriptome expression of *C. reinhardtii* when compared with the A15 and AR20 treatments. The number of upregulated transcripts for both treatments was larger than the downregulated transcripts. Despite the observed increase in the number of transcripts with dysregulated expression observed for A5, it is not possible to make conclusions about the concentration dependence of the responses, given the impossibility to extract RNA of good quality in the treatments with 100 and 200 mg L^−1^ nTiO_2_. Moreover, 36.7% of dysregulated genes had a fold change >4 or <−4 in 20 mg L^−1^ A5 treatment, suggesting that the exposure to the smaller size at the higher concentration of 20 mg L^−1^ nTiO_2_ resulted in a stronger dysregulation than other treatments.

Significantly dysregulated transcripts corresponded to biological pathways that include regulation of cell processes, energy metabolism (photosynthesis, carbohydrate metabolism), amino acid metabolism, stress and transport (Fig. S17, Table S10†), thus providing an indication about the cellular targets in *C. reinhardtii.* The number of dysregulated genes depended on the primary size of the tested nTiO_2_ and increased with concentration from 2 to 20 mg L^−1^. The exposure to two lower concentrations of nTiO_2_ resulted in significant changes in the adaptation of the nutrition pathways and metabolic production of energy as well as the induction of oxidative stress, and consequently, the activation of cellular protective response.

### Metabolic perturbation in *Chlamydomonas reinhardtii* exposed to nTiO_2_

#### Amino acid metabolism

**Fig. 7:**
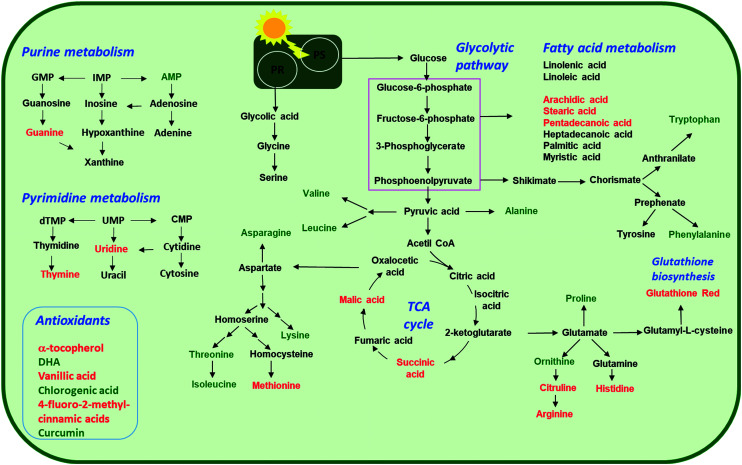
Overview of the proposed perturbations in metabolic pathways in green alga *C. reinhardtii* alga exposed to increasing concentrations (2, 20, 100 and 200 mg L^−1^) of nTiO_2_ with primary size of 5, 15 and 20 nm for 72 h. Accumulated or depleted responsive metabolites are present in red and green colors, respectively. PS: photosynthesis; PR: photorespiration. Only responsive metabolites were considered.

**Fig. 8:**
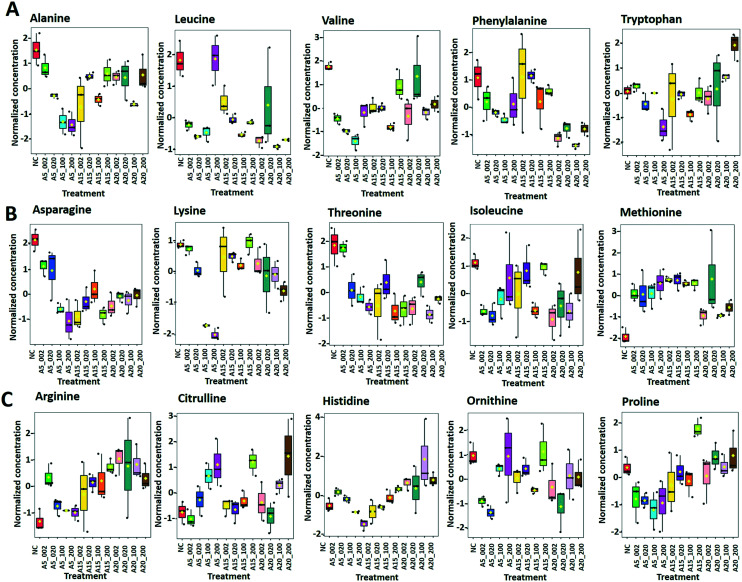
Box plots of relative abundance of (A) amino acids alanine, leucine, valine, phenylalanine and tryptophan, (B) aspartate-derived amino acids, and (C) α-ketoglutarate-derived amino acids responsive to treatment of *C. reinhardtii* with 2–200 mg L^−1^ nTiO_2_ with primary size of 5, 15 and 20 nm: treatments with: 5 nm nTiO_2_ 2 mg L^−1^ (A5_2), 20 mg L^−1^ (A5_20), 100 mg L^−1^ (A5_100) and 200 mg L^−1^ (A5_200); 15 nm nTiO_2_ 2 mg L^−1^ (A15_2), 20 mg L^−1^ (A15_20), 100 mg L^−1^ (A15_100) and 200 mg L^−1^ (A15_200); 20 nm nTiO_2_ (AR20): 2 mg L^−1^ (AR20_2), 20 mg L^−1^ (AR20_20), 100 mg L^−1^ (AR20_100) and 200 mg L^−1^ (AR20_200); negative control (NC). Data were row-wise normalized using probabilistic quotient normalization by reference groups, non-transformed and autoscaled.

Exposure to different nTiO_2_ induced a significant alteration in the abundance of 15 amino acids out of 21 amino acids that were quantified in *C. reinhardtii* (Fig. 7). *Alanine*, *leucine* and *valine* (Fig. 8A) produced from pyruvate^66^ were significantly reduced (*p* < 0.05) in A5 exposed cells. The reduction of these three metabolites was most important for the smallest size of the tested nTiO_2_ (A5) with a stronger decrease at higher exposure concentrations. *Leucine* concentrations were reduced in the treatments with A5 and A15, however no dependence on the exposure concentrations was observed for AR20. *Leucine*, a branched chain amino acid, serves as an oxidative phosphorylation energy source or as a detoxification pathway;^67^ its depletion may indicate that it was employed as a defense. Concentration dependent depletion of *valine* was induced only in A5, but not in A15 and AR20 exposures. The abundance of the amino acids derived from aspartate *asparagine*, *lysine* and *threonine* (Fig. 8) was significantly decreased with respect to the control with clear size and concentration dependences at A5 treatments. As *asparagine* is involved in ammonium assimilation in plants,^68^ it could be hypothesized that the capacity to acquire nitrogen compounds is partially inhibited by the nTiO_2_ treatments. The abundance of *isoleucine* was lower in nTiO_2_ cells than in the control, however the decrease was more substantial for treatments at lower nTiO_2_ concentration. *Methionine* accumulated in treatments with the three nTiO_2_ materials in comparison with the controls. The abundance of *methionine* significantly increased with increasing concentrations of A5, however a less clear concentration dependence was found in A15 and AR20 exposures. A clear dependence on the primary size of nTiO_2_ was observed in 100 and 200 mg L^−1^ treatments, with A5 being the most responsive. *Methionine* is the first amino acid to be translated in protein synthesis by initiating mRNA translation and is the precursor of essential bio-molecules through *S*-adenosylmethionine.^69^ Changes in its abundance could be used to provide an estimate of the rate of protein synthesis.^70^ Therefore, the accumulation of this metabolite suggests a significant acceleration of the rate of protein synthesis in A5 treatments.

*Arginine*, *citrulline* and *histidine* (Fig. 8C) biosynthesized from the TCA metabolite α-ketoglutarate^71^ increased in cells exposed to the three nTiO_2_. The abundance of *citrulline* increased with higher nTiO_2_ exposure concentrations, as well as the abundance of *arginine* in A15 and AR20 treatments.

*Ornithine* was depleted in nTiO_2_ treatments with respect to the control and the depletion was more pronounced for A5. *Histidine* and *proline* exhibited a more complex pattern with an increase in abundance with exposure concentration for A15, a decrease for A5 and lack of concentration dependence for AR20 treatments. As it is an amino acid needed for growth and development of algal cells,^72^ the accumulation reveals an impact on algal growth and cell development. In addition, *histidine* and *arginine* take part in deamination,^73^ showing that this process can be also altered. *Proline* plays an important role in osmo- and redox-regulation, metal chelation, and scavenging of free radicals induced by different metals in plants,^74,75^ therefore a decrease in abundance suggests a defensive response to oxidative stress.

Levels of aromatic amino acids, *phenylalanine* and *tryptophan*, derived from phosphoenolpyruvate were significantly reduced in a concentration dependent manner in A5 treatments only. Concentrations of two other amino acids, *glycine* and *serine*, measured in nTiO_2_ treatments and controls were comparable. As these amino acids are synthesized by the photorespiratory glycolate cycle in algae,^66^ the fact that no changes were observed in their concentration suggests no effect of nTiO_2_ on algal photorespiratory activity. These findings are in line with no observed changes in maximum photosynthetic yield and NPQ in most nTiO_2_ treatments (Fig. 2D and E), as well as with the lack of changes in the abundance of glucose which is a primary product of photosynthesis.

The metabolomics results are consistent with the strong dysregulation of the following transcripts involved in amino-acid metabolism. Four transcripts involved in amino acid metabolism were significantly affected by exposure to A5 at 20 mg L^−1^, but not to A15 and AR20 (Table S10†). The transcripts in genes coding for “phosphoserine aminotransferase” involved in the phosphorylated pathway of serine biosynthesis^76^ (Cre07.g331550.t1.2, FC −2.81, FDR 7.63 × 10^−9^) and “dihydrodipicolinate reductase” involved in l-lysine biosynthesis^77^ (Cre16.g656300.t1.3, FC −2.07, FDR 6.77 × 10^−7^), as well as peroxisomal 3-ketoacyl-CoA thiolase 3 in the isoleucine degradation pathway^78^ (Cre17.g723650.t1.2, FC −2.08, FDR 7.63 × 10^−9^), were down-regulated. The transcripts coding for fumarylacetoacetate hydrolase, which are involved in the final step of the tyrosine degradation pathway^79^ (Cre12.g549450.t1.2, FC 4.50, FDR 3.45 × 10^−10^), were up-regulated.

#### Nucleobase/tide/side metabolism

**Fig. 9:**
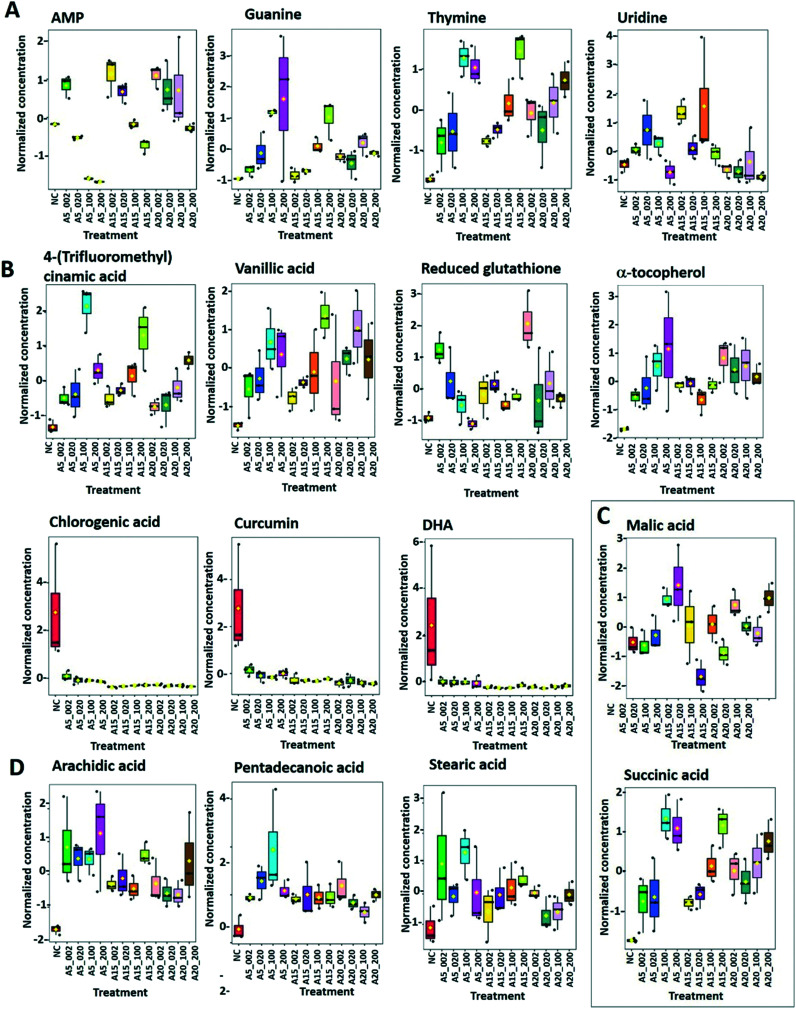
Box plots of relative abundance of the (A) responsive nucleobase/tides/sides of purine and pyrimidine metabolism; (B) responsive antioxidants; (C) carboxylic acids: malic and succinic acids; (D) fatty acids; arachidic, pentadecanoic and stearic acids in *C. reinhardtii* were treated with 2–200 mg L^−1^ nTiO_2_ with primary size of 5, 15 and 20 nm. Treatments with: 5 nm nTiO_2_ 2 mg L^−1^ (A5_2), 20 mg L^−1^ (A5_20), 100 mg L^−1^ (A5_100) and 200 mg L^−1^ (A5_200); 15 nm nTiO_2_ 2 mg L^−1^ (A15_2), 20 mg L^−1^ (A15_20), 100 mg L^−1^ (A15_100) and 200 mg L^−1^ (A15_200); 20 nm nTiO_2_ (AR20): 2 mg L^−1^ (AR20_2), 20 mg L^−1^ (AR20_20), 100 mg L^−1^ (AR20_100) and 200 mg L^−1^ (AR20_200); negative control (NC). Data were row-wise normalized using probabilistic quotient normalization by reference groups, non-transformed and autoscaled.

Among 15 nucleobases/tides/sides considered, 6 were quantified and 4 were statistically different from control treatments (*p* < 0.05) (Fig. 7 and 9A). The *pyrimidine* nucleotides/sides *uridine* and *thymine* accumulated in nTiO_2_ treatments. The *thymine* concentration increased with A5, A15 and AR20 concentrations. The *purine* metabolites (*AMP*, *guanine)* significantly increased in algae exposed to nTiO_2_ as compared with unexposed controls. *Guanine* levels rise with the exposure concentrations of A5 and A15; however, no concentration- or size-dependences were observed for AR20 treatments. A decrease of the abundance of AMP with the concentration of the nTiO_2_ was observed. Pyrimidine and purine nucleotides are structural elements of the nucleic acids DNA and RNA.^80^ Therefore, the present results could suggest an acceleration of DNA and RNA synthesis and turnover.

#### Antioxidant metabolism

Exposure to the nTiO_2_ with a primary size of 5 nm resulted in a significant increase of the abundance of *reduced glutathione* (GSH) in comparison with the control (Fig. 9B). Interestingly the increase was pronounced at lower concentrations of nTiO_2_ and decreased with an increase of the exposure concentration. This result suggests an activation of the defense mechanism against the oxidative stress induced by nTiO_2_, since GSH is central to redox control in the cell.^81^ Nevertheless, a significant generation of the ROS was found in *C. reinhardtii* exposed to nTiO_2_ (Fig. 2B). The above observation is consistent with the existing literature for metals and metal-containing NPs showing an increased level of GSH in green algae exposed to high concentrations of Cd^2+^ (ref. 82 and 83) or inorganic or monomethyl MeHg,^47^ whereas Cu exposure decreased GSH concentrations.^83,84^ Exposure of *P. melhamensis* to nAg and Ag ions also resulted in an accumulation of reduced GSH, which increased with the exposure time from 2 to 24 h.^38^

Dehydroascorbic acid (DHA, Fig. 9B), an oxidized form of ascorbic acid, was significantly depleted in the treatments with all three nTiO_2_ as compared with the control. However, no clear dependence on the concentration as a function of nTiO_2_ primary size or exposure concentration was found. Such a decrease of DHA probably corresponds to its consumption, *via* quenching of the ROS induced by nTiO_2_ exposure, to prevent the damage to important cellular components.

DHA depletion could suggest depletion of ascorbic acid and/or an acceleration of the recycling and regeneration of ascorbic acid used to eliminate ROS damage and enhance stress tolerance.^85^ Indeed, ascorbic acid participates in diverse cellular processes associated with photosynthetic functions and stress tolerance.^86^ For example, nAg and Ag^+^ induced an accumulation of ascorbic acid in *P. melhamensis*.^38^

α-Tocopherol accumulated in the all nTiO_2_ treatments in comparison to the control (Fig. 9B). The α-tocopherol abundance increased with the A5 and A15 exposure concentrations. However, no concentration dependence was found in AR20 treatments. α-Tocopherol is a major plastid prenyllipid antioxidant able to quench and scavenge singlet oxygen (^1^O_2_), as well as scavenge superoxide (O_2_˙^−^) and lipid radicals.^87^ The above observation is consistent with the literature showing an increase in the content of α- and γ-tocopherol in *C. reinhardtii* upon acclimation to chronic exposure to Cr_2_O_7_^2−^, Cd^2+^ and Cu^2+^.^88^ However, α-tocopherol concentration decreased significantly in *C. reinhardtii* exposed to pyrazolate.^89^

The abundance of polyphenols and phenolic acids, known as antioxidants, was also altered upon nTiO_2_ exposure. The concentrations of *vanillic* and *4-fluoro-2-methylcinnamic acids* (Fig. 9B) significantly increased in all three nTiO_2_ material treatments in an exposure concentration dependent manner. By contrast, chlorogenic acid (CGA) and curcumin were significantly depleted in the treatments with the three nTiO_2_. Similar depletion of these antioxidants was found in cyanobacterium *Nostoc sphaeroides* exposed to nAg and Ag^+^.^90^ CGA, the ester of caffeic acid and quinic acid, is known to play the role of an intermediate in lignin biosynthesis. The results indicate that the antioxidant defense system of *C. reinhardtii* was activated in nTiO_2_ exposure, which resulted in the alteration of the abundance of the ROS-scavenging metabolites to cope with the enhanced generation of ROS. No dependence in the changes of chlorogenic acid and curcumin levels with exposure concentration or primary size of nTiO_2_ was observed. Taken together, the above finding indicates that even at lower tested concentration of nTiO_2_ (*i.e.* 2 mg L^−1^) the antioxidant defense system of *C. reinhardtii* was affected, which resulted in an accumulation of the ROS-scavenging metabolites to cope with the enhanced generation of ROS (Fig. 2B). Nevertheless, the antioxidant system was overwhelmed in particularly for exposure to 100 and 200 mg L^−1^ nTiO_2_ as can be seen by the important percentage of cells experiencing oxidative stress.

The metabolomics results were also consistent with the transcriptomic profiling. A5 at 20 mg L^−1^ down-regulated the expression of transcripts of genes involved in reduction–oxidation reaction hemostasis, such as thioredoxin (Cre16.g656600.t1.2, TY2, FC −3.37, FDR 5.89 × 10^−10^) and glutathione peroxidase (Cre10.g458450.t1.3, GPX5, FC −5.07, and FDR 1.16 × 10^−8^). Indeed, thioredoxin is a key molecule in the response of *C. reinhardtii* to oxidative stress induced by dissolved metals and nanoparticles.^91,92^ In addition, the transcripts of genes associated with the stress response, *e.g.* “stress.abiotic.heat” such as chaperone DnaJ-domain superfamily protein were strongly up-regulated (Cre12.g488500.t1,2, FC 6.42, and FDR 1.82 × 10^−8^). The transcripts of genes related to oxidative stress tolerance coding for the cysteine-rich secretory protein family^93^ (Cre11.g467672.t1.1, FC 3.33, and FDR 4.96 × 10^−8^, Cre06.g278160.t1.1, FC 2.83, and FDR 1.24 × 10^−9^) and glycosyl hydrolase family protein^94^ (Cre13.g570700.t1.3, FC 2.36, and FDR 2.63 × 10^−10^) were also up-regulated. Moreover, genes involved in calcium-transporting ATPase 3, endoplasmic reticulum-type (ECA3), were also up-regulated indicating an increase of intracellular ROS production. These observations indicate an increased production of ROS induced by 20 mg L^−1^ A5 and subsequent modification of RedOx homeostasis balance in *C. reinhardtii*.

#### Carboxylic acid metabolism

Succinic acid concentration was significantly increased after treatments with all three nTiO_2_. The effect was more pronounced at higher concentrations of A5, A15 or AR20 (Fig. 9C). Treatments with A5, A15 and AR20 resulted in an increase of the concentration of malic acid, another TCA cycle intermediate. However the changes in the concentrations after AR20 treatments the abundance of the malic acid was lower than control at 2 and 20 mg L^−1^ nTiO_2_ treatments and increased at high concentrations (*i.e.*, 200 mg L^−1^.) This finding suggests that the key cellular metabolic pathway linking carbohydrate, fatty acids, and protein metabolism, was accelerated upon nTiO_2_ treatment probably to cope with an increase in energy production necessary for the manufacture of compounds needed to defend from nTiO_2_ stress. Alternatively, the accumulation of TCA cycle intermediates could be also a sign of their reduced conversion. The obtained results are consistent with the finding that the concentrations of succinic and malic acids increased significantly in *P. malhamensis* and *Chlorella vulgaris*^40^ exposed to Ag^+^ and nAg, as well as in *C. reinhardtii* exposed to high concentration of inorganic Hg,^47^ as well as the level of malic acid in *Scenedesmus obliquus* exposed to nAg.^39^ By contrast, a decrease in the TCA intermediates was observed in the diatom *T. flocculosa* exposed to high Cu concentrations.^95^

#### Fatty acid metabolism

Among the 8 fatty acids that were measured, 3 saturated acids (*i.e.* pentadecylic acid (pentadecanoic acid, 15 : 0), stearic (octadecanoic acid, 18 : 0), arachidic (icosanoic acid, 20 : 0)) accumulated in cells exposed to nTiO_2_ (Fig. 7 and 9D). However, no clear dependences on nTiO_2_ primary size or exposure concentrations were observed. These results suggest that algae exposed to nTiO_2_ modify the membrane fluidity to make it more tolerant to oxidation, thus preserving membrane integrity under oxidative stress conditions.^96^ Indeed, saturated acids and in particular palmitic acid are known to be less prone to oxidation than other fatty acids.^96^ Interestingly, changes in the abundance of unsaturated linoleic and linolenic acids were not significantly different from the unexposed control (all *p* > 0.05) with the exception of treatments with 2 mg L^−1^ A5, A15 and AR20. These findings suggest no significant unsaturation of the lipid membranes and alteration of integrity of lipid membranes. Indeed, this finding agrees with the relatively low percentage of cells with the affected membrane permeability found by PI in nTiO_2_ treatments (Fig. 2C).

Similar accumulation of fatty acids has been observed in algae under toxic metal stress.^97^ However, the concentrations of arachidic and stearic acids decreased in *P. malhamensis* treated with nAg.^38^ nAg and Ag^+^ also induced a reduction in the abundance of monounsaturated and polyunsaturated fatty acids of *Chlorella vulgaris*.^98^ Plants were also shown to regulate the composition of fatty acids in the membrane to rebuild membrane integrity, as was shown for cucumber leaves exposed to nAg and Ag^+^.^99^

### Influence of nTiO_2_ treatments on nutrient transport

Exposure to 20 mg L^−1^ A5 up-regulated the expression of most transcripts involved in transport, adenosine triphosphate (ATP) binding cassette (ABC) transporters, and metal transporters. Among the dysregulated ABC transporters, the multidrug resistance-associated protein 12 (Cre10.g458450.t1.3, MRP12, FC 7.38, FDR 6.40 × 10^−10^) and P-loop containing nucleoside triphosphate hydrolase superfamily protein (Cre10.g444700.t1.1, FC 7.96, FDR 6.71 × 10^−11^) were strongly up-regulated suggesting the involvement of the cellular mechanism in metal detoxification in algal.^100,101^ Several zinc (Zn)-regulated transporters, iron (Fe)-regulated transporter-like proteins (ZIP), transporters of Fe and Zn, and Zn transporters were also up-regulated. These regulations suggest the impact of A5 on the global nutrition of the microalgal.^102^ A15 and AR20 significantly dysregulated similar numbers and categories of genes (Table S10†), mostly involved in the ABC transporters and metal transporters at both concentrations.

Overall the present results revealed that 72 h exposure of green alga to nTiO_2_ with different primary sizes altered the metabolism of amino acids, nucleotides, fatty acids, tricarboxylic acids and antioxidants, and resulted in a disturbance in a global nutrition in a concentration- and primary size-dependent way, despite the formation of micrometer-size aggregates and their sedimentation. These disturbances were consistent with the observed oxidative stress and growth inhibition observations. However, it is worth noting that the bioassays were performed at concentrations of nTiO_2_ much higher than those typically found in the freshwater environment. As the metabolic perturbations and modes of toxic action may not be the same at a low or high exposure concentration, an extrapolation of the present observations to natural environment conditions is limited. In addition, the exposure of 72 h corresponding to chronic toxicity tests for algae was selected to gain a mechanistic understanding of the adverse outcome in an advanced time frame relative to when the physiological responses are typically assessed. However, the prolonged exposure could trigger other modes of action over time and different defense/detoxification mechanisms.^35^ Despite these limitations, the study provides a unique and novel biological insight into the perturbation of the algal metabolism by nTiO_2_ and their aggregates and potentially directs further mechanistic studies with other phytoplankton species and nanomaterials.

## Conclusion

The present exploratory study provides, for the first-time, evidence of the metabolic perturbations in green alga *C. reinhardtii* exposed to increasing concentration of nTiO_2_ with different primary sizes. The results revealed that despite the important aggregation and sedimentation, the exposure to increasing concentrations of nTiO_2_ with primary sizes of 5, 15 and 20 nm altered the abundance of metabolites involved in various pathways corresponding to amino acid, nucleotides, fatty acid, TCA and antioxidant metabolism. Most of the responsive metabolites were common for all the treatments, however the intensity of the response and the existence or not of concentration- and nanoparticle primary size-dependences differed among the treatments. The metabolomics results correlate well with the transcriptomics and physiological results and confirmed that oxidative stress is a major toxicity mechanism for nTiO_2_. The findings contribute to an improvement of knowledge concerning the molecular basis of these perturbations and thus to the understanding of environmental implications of one of the most used engineered nanomaterials.

## Author contributions

V. I. S., W. L. and M. T. conceived and designed the study. M. T. performed the nTiO_2_ characterization, bioassays for physiological response assessment, exposure assays for metabolomics, analyzed the physiological response data and provided interpretations, and wrote this part of the manuscript; W. L. performed exposure for transcriptomics, RNA extraction, and data processing, and wrote the transcriptomics part of the manuscript; W. W. L. performed the LC-MS measurements and data processing; V. I. S. performed analysis and interpretation of metabolomics results, wrote the metabolomics part of the manuscript, and overviewed the overall study. A. A. K. took part in the data interpretation, manuscript writing, and overviewed the overall study. All the authors critically commented and revised the manuscript. All the authors have approved the paper submission.

## Conflicts of interest

The authors declare no competing interests.

## Supplementary Material

EN-009-D2EN00260D-s001
