# Supplementary material for: Metabolic alterations in alga Chlamydomonas reinhardtii exposed to nTiO2 materials
Source: Environ Sci Nano. 2022 Jul 1;9(8):2922–38. doi: 10.1039/d2en00260d (PMC9367718; doi:10.1039/d2en00260d)
Supplement: EN-009-D2EN00260D-s001 [file EN-009-D2EN00260D-s001.pdf]

Electronic Supplementary Material (ESI) for Environmental Science: Nano.
This journal is © The Royal Society of Chemistry 2022

Supplementary information

Metabolic alterations in alga *Chlamydomonas reinhardtii* exposed to $nTiO_2$ materials

Wei Liu[a], Mengting Li[a], Weiwei Li[b], Arturo A. Keller[b]. Vera I. Slaveykova[a,*]

[a] University of Geneva. Faculty of Sciences. Earth and Environment Sciences. Department F.-A. Forel for Environmental and Aquatic Sciences. Environmental Biogeochemistry and Ecotoxicology. Uni Carl Vogt. 66 Blvd Carl-Vogt. CH 1211 Geneva. Switzerland

[b] Bren School of Environmental Science & Management. University of California. Santa Barbara. California 93106-5131. United States

*Corresponding author: vera.slaveykova@unige.ch

List of Tables

Table S1. Characteristics of $nTiO_2$ materials as provided by the manufacturer

Table S2. NanoString probes design

Table S3. Important features identified by One-way ANOVA and Fisher's post-hoc analysis in *C. reinhardtii* exposed to 5 nm nanoTiO_2

Table S4. Important features identified by One-way ANOVA and Fisher's post-hoc analysis in *C. reinhardtii* exposed to 15 nm nanoTiO_2

Table S5. Important features identified by One-way ANOVA and Fisher's post-hoc analysis in *C. reinhardtii* exposed to 20 nm nanoTiO_2

Table S6. Important features identified by One-way ANOVA and Fisher's post-hoc analysis) in *C. reinhardtii* exposed to 2 mg L^{-1} of nanoTiO_2 of different primary size: 5nm (A5), 15nm (A15) and 20nm (AR20).

Table S7. Important features identified by One-way ANOVA and Fisher's post-hoc analysis) in *C. reinhardtii* exposed to 20 mg L^{-1} of nanoTiO_2 of different primary size: 5nm (A5), 15nm (A15) and 20nm (AR20)

Table S8. Important features identified by One-way ANOVA and Fisher's post-hoc analysis) in *C. reinhardtii* exposed to 100 mg L^{-1} of nanoTiO_2 of different primary size: 5nm (A5), 15nm (A15) and 20nm (AR20)

Table S9. Important features identified by One-way ANOVA and Fisher's post-hoc analysis) in *C. reinhardtii* exposed to 200 mg L^{-1} of nanoTiO_2 of different primary size: 5nm (A5), 15nm (A15) and 20nm (AR20)

Table S10. Number of significantly dysregulated genes in metabolic pathways (MapMan) in *C. reinhardtii* after 72 h exposure to A5, A15 and AR20 at 2 and 20 mgL^{-1}

List of Figures

Figure S1. PCA analysis of metabolic response of *C. reinhardtii* treated with $nTiO_2$ of increasing concentrations.

Figure S2. PCA analysis of metabolic response of *C. reinhardtii* treated with $nTiO_2$ of different primary sizes.

Figure S3. Variable Importance in the Projection (VIP) scores from PLS-DA analysis of discriminating metabolites between unexposed controls and treatments with A5, A15 and AR20 of increasing concentrations.

Figure S4. VIP scores from PLS-DA analysis of discriminating metabolites between unexposed controls and A5, A15 and AR20 treatments of different size at given concentration.

Figure S5. Heat map presentation of the significantly altered metabolites *in C. reinhardtii* treated with increasing concentrations of $nTiO_2$ with primary size of 5 nm.

Figure S6. Heat map presentation of the significantly altered metabolites *in C. reinhardtii* treated with increasing concentrations of $nTiO_2$ with primary size of 15 nm.

Figure S7. Heat map presentation of the significantly altered metabolites *in C. reinhardtii* treated with increasing concentrations of $nTiO_2$ with primary size of 20 nm.

Figure S8. Heat map presentation of the significantly altered metabolites in *C. reinhardtii* treated with 2 mgL^{-1} $nTiO_2$ at different primary sizes.

Figure S9. Heat map presentation of the significantly altered metabolites in *C. reinhardtii* treated with 20 mgL^{-1} $nTiO_2$ at different primary sizes.

Figure S10. Heat map presentation of the significantly altered metabolites in *C. reinhardtii* treated with 100 mgL^{-1} $nTiO_2$ at different primary sizes.

Figure S11. Heat map presentation of the significantly altered metabolites in *C. reinhardtii* treated with 200 mgL^{-1} $nTiO_2$ at different primary sizes.

Figure S12. Pathway analysis for metabolites with altered abundance in *C. reinhardtii* exposed to different concentrations of $nTiO_2$ with different size (A) 5nm, (B) 15nm and (C) 20 nm.

Figure S13. Pathway analysis for metabolites with altered abundance in *C. reinhardtii* exposed to different concentrations of $nTiO_2$ with size of 5, 15 and 20 nm (A) 2 mgL^{-1}, (B) 20 mgL^{-1}, (C) 100 $mgL^{-1}$and (D) 200 $mgL^{-1}$$nTiO_2$

Figure S14. PCA analysis of transcriptome response of *C. reinhardtii* treated with 2 and 20 mgL^{-1} $nTiO_2$.

Figure S15. PCA analysis of transcriptome response of *C. reinhardtii* treated with $nTiO_2$. different primary sizes.

Figure S16. Distribution of number of significantly dysregulated transcripts corresponding to pathways in *C. reinhardtii* exposed to A5, A15 and AR20 at 2 mg L^{-1}, and 20 mg L^{-1} concentrations.

Table S1. Properties of $nTiO_2$ material as dry powder as provided by the manufacturer https://www.nanoamor.com/ and also hydrodynamic diameter and zeta potential measured in the suspensions of MilliQ water measured in our lab.

**Material**	**A5**	**A15**	**AR20**
Composite	anatase	anatase	anatase (80-90%) /rutile (10-20%)
Average particle size (nm)	5	15	20
Purity (%)	99.8	99	99+
Specific Surface Area (m^2 g^{-1})	150-300	~240	≥ 5
Bulk density (g cm^{-3})	0.25-0.3	0.04-0.06	0.4-0.5
TEM images	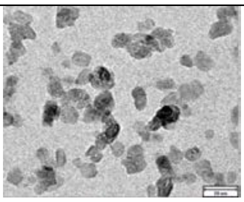	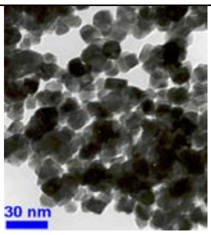	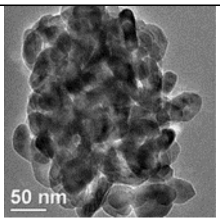

Table S2. NanoString probes design.

Accession Number	*Target Region*	*Accession Number*	*Target Region*
Cre01_g000150_t1_2.1	2756-2855	*Cre08_g360400_t1_3.1*	5005-5104
Cre01_g014350_t1_2.1	1105-1204	*Cre08_g360500_t1_3.1*	101-200
Cre01_g019950_t1_2.1	436-535	*Cre08_g362900_t1_3.1*	700-799
Cre01_g027764_t1_2.1	2207-2306	*Cre08_g365900_t1_2.1*	879-978
Cre01_g036850_t1_2.1	303-402	*Cre08_g367600_t1_2.1*	1472-1571
Cre01_g039850_t1_3.1	2624-2723	*Cre08_g369720_t1_1.1*	3391-3490
Cre01_g066187_t1_1.1	1824-1923	*Cre09_g392729_t1_1.1*	2032-2131
Cre02_g077350_t1_2.1	218-317	*Cre09_g396600_t1_1.1*	2298-2397
Cre02_g080650_t1_2.1	2901-3000	*Cre09_g402500_t1_2.1*	1770-1869
Cre02_g080900_t1_2.1	1033-1132	*Cre09_g407501_t1_2.1*	3304-3403
Cre02_g081400_t1_2.1	2626-2725	*Cre09_g410050_t1_2.1*	4700-4799
Cre02_g083800_t1_2.1	244-343	*Cre09_g410100_t1_2.1*	4075-4174
Cre02_g090850_t1_3.1	3338-3437	*Cre09_g416200_t1_2.1*	109-208
Cre02_g095069_t1_1.1	947-1046	*Cre09_g417150_t1_2.1*	142-241
Cre02_g095076_t1_1.1	1516-1615	*Cre10_g423450_t1_2.1*	137-236
Cre02_g095151_t1_1.1	3317-3416	*Cre10_g424775_t1_1.1*	3799-3898
Cre02_g095200_t1_2.1	1684-1783	*Cre10_g429800_t1_2.1*	199-298
Cre02_g097800_t1_3.1	2306-2405	*Cre10_g444700_t1_1.1*	3340-3439
Cre02_g109600_t1_2.1	497-596	*Cre10_g458450_t1_3.1*	935-1034
Cre02_g115350_t1_2.1	841-940	*Cre10_g458500_t1_2.1*	1795-1894
Cre02_g117500_t1_2.1	2275-2374	*Cre11_g467672_t1_1.1*	1764-1863
Cre03_g146527_t1_1.1	2048-2147	*Cre12_g488500_t1_2.1*	397-496
Cre03_g148250_t1_3.1	11670-11769	*Cre12_g514850_t1_2.1*	2683-2782
Cre03_g149300_t1_3.1	93-192	*Cre12_g530350_t1_3.1*	2030-2129
Cre03_g155001_t1_1.1	4517-4616	*Cre12_g530400_t1_2.1*	1628-1727
Cre03_g160800_t1_2.1	606-705	*Cre12_g533950_t1_3.1*	1053-1152
Cre03_g162333_t1_1.1	5637-5736	*Cre12_g534800_t1_1.1*	2211-2310
Cre03_g164600_t1_2.1	3735-3834	*Cre12_g542300_t1_2.1*	1901-2000
Cre03_g173800_t1_2.1	1740-1839	*Cre12_g545850_t1_2.1*	1795-1894
Cre03_g181500_t1_2.1	912-1011	*Cre12_g549450_t1_2.1*	1220-1319
Cre03_g185250_t1_2.1	2025-2124	*Cre12_g553250_t1_2.1*	2104-2203
Cre03_g185300_t1_2.1	1885-1984	*Cre13_g565260_t1_1.1*	3548-3647

Cre03_g195050_t1_2.1	3142-3241	*Cre13_g570350_t1_3.1*	2674-2773
Cre03_g195600_t1_2.1	123-222	*Cre13_g570700_t1_3.1*	2822-2921
Cre03_g199800_t1_1.1	1714-1813	*Cre13_g574500_t1_3.1*	1202-1301
Cre03_g205850_t1_2.1	2041-2140	*Cre14_g613600_t1_2.1*	1785-1884
Cre04_g215150_t1_2.1	2133-2232	*Cre14_g629650_t1_3.1*	2837-2936
Cre04_g217913_t1_1.1	383-482	*Cre14_g629960_t1_1.1*	338-437
Cre04_g227450_t1_2.1	218-317	*Cre16_g650200_t1_3.1*	1648-1747
Cre05_g233304_t1_1.1	3211-3310	*Cre16_g656300_t1_3.1*	1616-1715
Cre05_g233900_t1_2.1	1143-1242	*Cre16_g656600_t1_2.1*	1338-1437
Cre05_g237400_t1_2.1	1614-1713	*Cre16_g669525_t1_1.1*	652-751
Cre05_g248300_t1_3.1	2733-2832	*Cre16_g683450_t1_2.1*	2058-2157
Cre06_g258733_t2_1.1	993-1092	*Cre16_g688526_t1_1.1*	5054-5153
Cre06_g260250_t1_2.1	100-199	*Cre16_g692800_t1_3.1*	235-334
Cre06_g260450_t1_2.1	2402-2501	*Cre16_g694500_t1_3.1*	399-498
Cre06_g263550_t1_2.1	989-1088	*Cre16_g695100_t1_3.1*	2345-2444
Cre06_g263950_t1_3.1	2288-2387	*Cre17_g698850_t1_3.1*	127-226
Cre06_g271400_t1_3.1	1-100	*Cre17_g703176_t1_1.1*	50-149
Cre06_g276050_t1_2.1	1493-1592	*Cre17_g707700_t1_2.1*	1476-1575
Cre06_g278142_t1_1.1	158-257	*Cre17_g720400_t1_3.1*	4822-4921
Cre06_g278160_t1_1.1	1693-1792	*Cre17_g723650_t1_2.1*	1683-1782
Cre06_g296400_t1_2.1	1164-1263	*Cre17_g732300_t1_2.1*	2034-2133
Cre07_g315200_t1_3.1	5117-5216	*Cre17_g743547_t1_1.1*	1718-1817
Cre07_g319500_t1_3.1	2820-2919	*Cre18_g749697_t1_1.1*	212-311
Cre07_g331550_t1_2.1	1516-1615	***House-Keeping genes***	
Cre07_g339104_t1_2.1	6333-6432	*Cre06_g260950_t1_2.1*	11-110
Cre07_g343933_t1_1.1	5563-5662	*Cre06_g272950_t1_1.1*	215-314
Cre07_g355100_t1_2.1	2642-2741	*Cre08_g370550_t1_1.1*	72-171
Cre07_g355150_t1_3.1	2149-2248	*Cre09_g411100_t1_2.1*	298-397
Cre07_g357300_t1_2.1	694-793	*Cre12_g519180_t1_1.1*	276-375
Cre08_g360050_t1_3.1	4664-4763	*Cre12_g519450_t1_1.1*	1358-1457

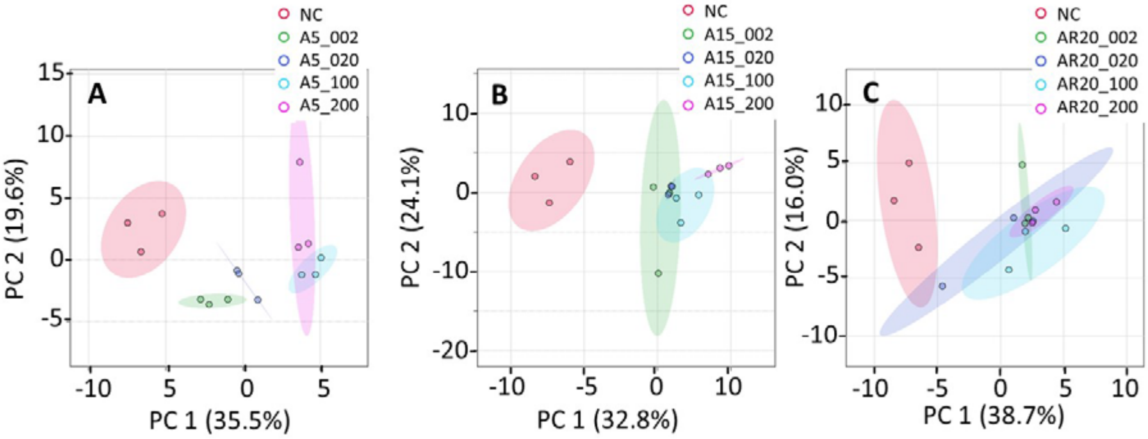

Figure S1. PCA analysis of metabolic response of *C. reinhardtii* treated with increasing concentrations three $nTiO_2$ materials. Treatments with: (A) 5 nm $nTiO_2$ 2 mg L^{-1} (A5_2), 20 mg L^{-1} (A5_20), 100 mg L^{-1} (A5_100) and 200 mg L^{-1} (A5_200); (B) 15 nm $nTiO_2$ 2 mg L^{-1} (A15_2), 20 mg L^{-1} (A15_20), 100 mg L^{-1} (A15_100) and 200 mg L^{-1} (A15_200); (C) 20 nm $nTiO_2$ (AR20): 2 mg L^{-1} (AR20_2), 20 mg L^{-1} (AR20_20), 100 mg L^{-1} (AR20_100) and 200 mg L^{-1} (AR20_200). Data were row-wise normalized using probabilistic quotient normalization by reference groups, non-transformed and autoscaled. The score plots and heatmap are generated by MetaboAnalyst 5.0 (https://www.metaboanalyst.ca/).

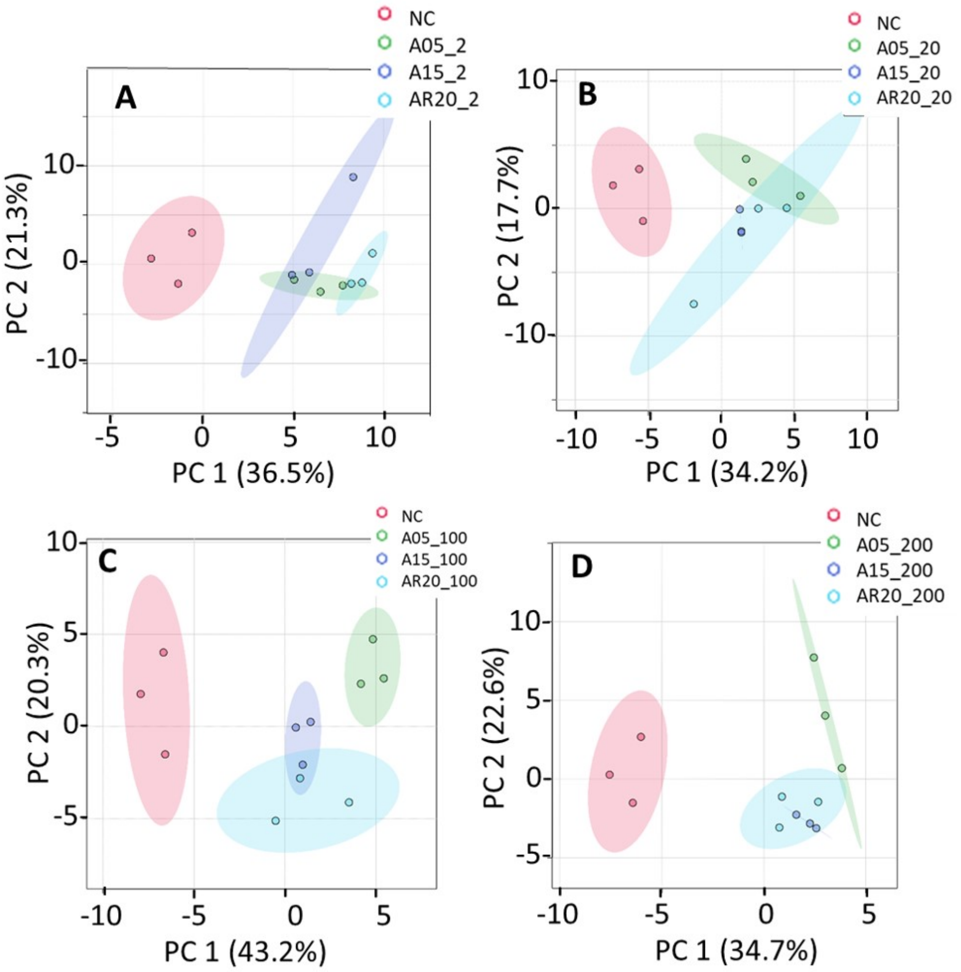

Figure S2. PCA analysis of metabolic response of *C. reinhardtii* treated with $nTiO_2$ materials of different primary size. Treatments with: (A) 2 mg L^{-1} of $nTiO_2$ with size of 5nm (A5_002), 15 nm (A15_002) and 20 nm (AR20_002); (B) 20 mg L^{-1} of $nTiO_2$ with size of 5nm (A5_020), 15 nm (A15_020) and 20 nm (AR20_020); (C) 100 mg L^{-1} of $nTiO_2$ with size of 5nm (A5_100), 15 nm (A15_100) and 20 nm (AR20_100); (D) 200 mg L^{-1} of $nTiO_2$ with size of 5nm (A5_200), 15 nm (A15_200) and 20 nm (AR20_200). Data were row-wise normalized using probabilistic quotient normalization by reference groups, non-transformed and autoscaled. The score plots is generated by MetaboAnalyst 5.0 (https://www.metaboanalyst.ca/)

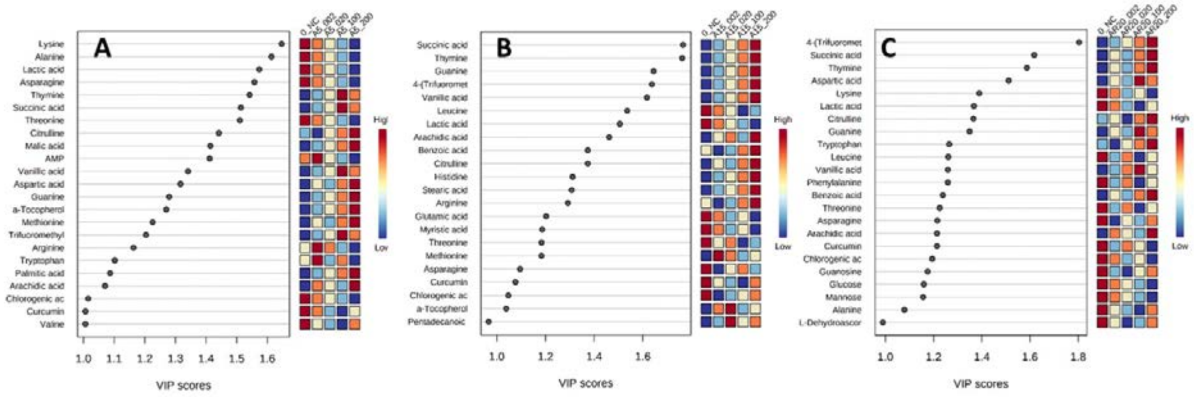

Figure S3. Variable Importance in the Projection (VIP) scores from PLS-DA analysis of discriminating metabolites between unexposed controls and A5. A15 and AR20 treatments. The colored boxes on the right indicate the relative concentrations of the corresponding metabolite in each group under study. Treatments with (A) 5 nm nTiO_2 (A5): 2 mg L^{-1} (A5_2), 20 mg L^{-1} (A5_20),100 mg L^{-1} (A5_100) and 200 mg L^{-1} (A5_200); (B) 15 nm nTiO_2 (A15): 2 mg L^{-1} (A15_2), 20 mg L^{-1} (A15_20), 100 mg L^{-1} (A15_100) and 200 mg L^{-1} (A15_200); (C) 20 nm nTiO_2 (AR20): 2 mg L^{-1} (AR20_2),20 mg L^{-1} (AR20_20), 100 mg L^{-1} (AR20_100) and 200 mg L^{-1} (AR20_200). Only metabolites with a VIP >1, regarded as significant are present.

Table S3. Important features identified by One-way ANOVA and Fisher's post-hoc analysis ($p < 0.05$) in *C. reinhardtii* exposed to 5 nm $nTiO_2$ (A5): 2 mg L^{-1} (A5_2), 20 mg L^{-1} (A5_20), 100 mg L^{-1} (A5_100) and 200 mg L^{-1} (A5_200). Data were row-wise normalized using probabilistic quotient normalization by reference groups. non-transformed and autoscaled.

Metabolite	f.value	p.value	-log10(p)	FDR	Fisher's LSD
AMP	174.58	0	8.4782	0	A5_002 - A5_020; A5_002 - A5_100; A5_002 - A5_200; A5_002 - NC; A5_020 - A5_100; A5_020 - A5_200; NC - A5_020; NC - A5_100; NC - A5_200
Lysine	165.54	0	8.3646	0	A5_002 - A5_020; A5_002 - A5_100; A5_002 - A5_200; A5_020 - A5_100; A5_020 - A5_200; NC - A5_020; NC - A5_100; NC - A5_200
Valine	59.45	0	6.2072	0	A5_002 - A5_020; A5_002 - A5_100; NC - A5_002; A5_200 - A5_020; NC - A5_020; A5_200 - A5_100; NC - A5_100; NC - A5_200
Lactic acid	34.977	0	5.1258	0.0001	A5_002 - A5_100; A5_002 - A5_200; NC - A5_002; A5_020 - A5_100; A5_020 - A5_200; NC - A5_020; NC - A5_100; NC - A5_200
Guanosine	29.288	0	4.7729	0.0002	A5_100 - A5_002; A5_200 - A5_002; NC - A5_002; A5_100 - A5_020; A5_200 - A5_020; NC - A5_020; NC - A5_100; NC - A5_200
Alanine	22.287	0.0001	4.2414	0.0004	A5_002 - A5_020; A5_002 - A5_100; A5_002 - A5_200; A5_020 - A5_100; A5_020 - A5_200; NC - A5_020; NC - A5_100; NC - A5_200
Leucine	21.9	0.0001	4.2079	0.0004	A5_200 - A5_002; NC - A5_002; A5_200 - A5_020; NC - A5_020; A5_200 - A5_100; NC - A5_100
Arginine	20.655	0.0001	4.0963	0.0005	A5_002 - A5_100; A5_002 - A5_200; A5_002 - NC; A5_020 - A5_100; A5_020 - A5_200; A5_100 - A5_200; NC - A5_200
Trifuoromethyl cinnamic acid	17.712	0.0002	3.8074	0.0009	A5_100 - A5_002; A5_100 - A5_020; A5_100 - A5_200; A5_100 - NC; A5_200 - NC
Thymine	14.26	0.0004	3.4111	0.0019	A5_100 - A5_002; A5_200 - A5_002; A5_100 - A5_020; A5_200 - A5_020; A5_020 - NC; A5_100 - NC; A5_200 - NC
Threonine	14.026	0.0004	3.3815	0.0019	A5_002 - A5_020; A5_002 - A5_100; A5_002 - A5_200; NC - A5_020; NC - A5_100; NC - A5_200
Asparagine	12.765	0.0006	3.2144	0.0025	A5_002 - A5_100; A5_002 - A5_200; A5_020 - A5_100; A5_020 - A5_200; NC - A5_100; NC - A5_200
Succinic acid	12.381	0.0007	3.1609	0.0027	A5_100 - A5_002; A5_200 - A5_002; A5_100 - A5_020; A5_200 - A5_020; A5_100 - NC; A5_200 - NC
Histidine	11.504	0.0009	3.0334	0.0033	A5_002 - A5_020; A5_002 - A5_100; A5_002 - A5_200; A5_002 - NC; A5_020 - NC
Benzoic acid	9.9106	0.0017	2.7809	0.0055	A5_100 - A5_002; A5_002 - NC; A5_100 - A5_020; A5_020 - NC; A5_100 - A5_200; A5_100 - NC; A5_200 - NC
Glutathione	8.8584	0.0025	2.5967	0.0079	A5_002 - A5_100; A5_002 - A5_200; A5_002 - NC; A5_020 - A5_200; A5_020 - NC
Citrulline	7.9634	0.0037	2.4269	0.011	A5_100 - A5_002; A5_200 - A5_002; A5_200 - A5_020; A5_100 - NC; A5_200 - NC
Tryptophan	7.4519	0.0047	2.3237	0.0132	A5_002 - A5_200; A5_020 - A5_200; A5_100 - A5_200; NC - A5_200
Malic acid	6.8156	0.0065	2.1881	0.0171	A5_100 - A5_002; A5_200 - A5_002; A5_200 - A5_020; A5_100 - NC; A5_200 - NC
Methionine	6.6452	0.0071	2.1504	0.0177	A5_002 - NC; A5_020 - NC; A5_100 - NC; A5_200 - NC
Ornithine	5.6475	0.0121	1.9155	0.0289	A5_200 - A5_002; NC - A5_002; A5_100 - A5_020; A5_200 - A5_020; NC - A5_020
Vanillic acid	5.0846	0.0169	1.7713	0.0385	A5_100 - A5_002; A5_020 - NC; A5_100 - NC; A5_200 - NC

Table S4. Important features identified by One-way ANOVA and Fisher's post-hoc analysis ($p < 0.05$) in *C. reinhardtii* exposed to 15 nm $nTiO_2$ (A15): 2 mg L^{-1} (A15_2), 20 mg L^{-1} (A15_20), 100 mg L^{-1} (A15_100) and 200 mg L^{-1} (A15_200). Data were row-wise normalized using probabilistic quotient normalization by reference groups. non-transformed and autoscaled.

Column1	f.value	p.value	-log10(p)	FDR	Fisher's LSD
Guanosine	70.45	0	6.5598	0	0_NC - A15_002; 0_NC - A15_020; 0_NC - A15_100; 0_NC - A15_200; A15_100 - A15_002; A15_200 - A15_002; A15_200 - A15_020; A15_200 - A15_100
Methionine	40.622	0	5.4271	0.0001	A15_002 - 0_NC; A15_020 - 0_NC; A15_100 - 0_NC; A15_200 - 0_NC
Thymine	28.806	0	4.7402	0.0003	A15_002 - 0_NC; A15_020 - 0_NC; A15_100 - 0_NC; A15_200 - 0_NC; A15_100 - A15_002; A15_200 - A15_002; A15_200 - A15_020; A15_200 - A15_100
Succinic acid	26.891	0	4.6052	0.0003	A15_002 - 0_NC; A15_020 - 0_NC; A15_100 - 0_NC; A15_200 - 0_NC; A15_100 - A15_002; A15_200 - A15_002; A15_100 - A15_020; A15_200 - A15_020; A15_200 - A15_100
Lactic acid	24.172	0	4.3978	0.0004	0_NC - A15_002; 0_NC - A15_020; 0_NC - A15_100; 0_NC - A15_200
Leucine	22.328	0.0001	4.2449	0.0005	0_NC - A15_002; 0_NC - A15_020; 0_NC - A15_100; 0_NC - A15_200; A15_002 - A15_100; A15_002 - A15_200
Valine	20.51	0.0001	4.083	0.0006	0_NC - A15_002; 0_NC - A15_020; 0_NC - A15_100; 0_NC - A15_200; A15_002 - A15_100; A15_200 - A15_002; A15_020 - A15_100; A15_200 - A15_020; A15_200 - A15_100
AMP	19.597	0.0001	3.9968	0.0006	A15_002 - 0_NC; A15_020 - 0_NC; A15_002 - A15_100; A15_002 - A15_200; A15_020 - A15_100; A15_020 - A15_200
Guanine	19.486	0.0001	3.9861	0.0006	A15_100 - 0_NC; A15_200 - 0_NC; A15_100 - A15_002; A15_200 - A15_002; A15_100 - A15_020; A15_200 - A15_020; A15_200 - A15_100
Asparagine	17.144	0.0002	3.747	0.0009	0_NC - A15_002; 0_NC - A15_020; 0_NC - A15_100; 0_NC - A15_200; A15_100 - A15_002; A15_100 - A15_200
a-Tocopherol	16.022	0.0002	3.6224	0.0011	A15_002 - 0_NC; A15_020 - 0_NC; A15_100 - 0_NC; A15_200 - 0_NC; A15_020 - A15_100
Arachidic acid	13.828	0.0004	3.3561	0.0018	A15_002 - 0_NC; A15_020 - 0_NC; A15_100 - 0_NC; A15_200 - 0_NC; A15_200 - A15_002; A15_200 - A15_020; A15_200 - A15_100
Citrulline	10.94	0.0011	2.9473	0.0043	A15_200 - 0_NC; A15_200 - A15_002; A15_200 - A15_020; A15_200 - A15_100
4-(Trifuoromethyl)cinnamic acid	10.65	0.0013	2.9016	0.0045	A15_020 - 0_NC; A15_100 - 0_NC; A15_200 - 0_NC; A15_200 - A15_002; A15_200 - A15_020; A15_200 - A15_100
Vanillic acid	10.442	0.0014	2.8683	0.0045	A15_020 - 0_NC; A15_100 - 0_NC; A15_200 - 0_NC; A15_200 - A15_002; A15_200 - A15_020; A15_200 - A15_100
Benzoic acid	8.3909	0.0031	2.5097	0.0097	A15_200 - 0_NC; A15_200 - A15_002; A15_200 - A15_020; A15_200 - A15_100
Proline	5.2705	0.0151	1.82	0.0445	A15_200 - 0_NC; A15_200 - A15_002; A15_200 - A15_020; A15_200 - A15_100
Threonine	5.1403	0.0164	1.786	0.0455	0_NC - A15_002; 0_NC - A15_100; 0_NC - A15_200
Curcumin	4.9296	0.0186	1.7299	0.049	0_NC - A15_002; 0_NC - A15_020; 0_NC - A15_100; 0_NC - A15_200

Table S5. Important features identified by One-way ANOVA and Fisher's post-hoc analysis ($p < 0.05$) in *C. reinhardtii* exposed to 20 nm $nTiO_2$ (AR20): 2 mg L^{-1} (AR20_2), 20 mg L^{-1} (AR20_20), 100 mg L^{-1} (AR20_100) and 200 mg L^{-1} (AR20_200). Data were row-wise normalized using probabilistic quotient normalization by reference groups. non-transformed and autoscaled.

Column1	f.value	p.value	-log10(p)	FDR	Fisher's LSD
Guanosine	30.91	0	4.8795	0.0007	0_NC - AR20_002; 0_NC - AR20_020; 0_NC - AR20_100; 0_NC - AR20_200
Asparagine	21.249	0.0001	4.1503	0.0018	0_NC - AR20_002; 0_NC - AR20_020; 0_NC - AR20_100; 0_NC - AR20_200
Phenylalanine	17.707	0.0002	3.8069	0.0026	0_NC - AR20_002; 0_NC - AR20_020; 0_NC - AR20_100; 0_NC - AR20_200
Threonine	14.467	0.0004	3.4371	0.0046	0_NC - AR20_002; 0_NC - AR20_020; 0_NC - AR20_100; 0_NC - AR20_200; AR20_020 - AR20_002; AR20_020 - AR20_100
Succinic acid	12.176	0.0007	3.1317	0.0072	AR20_002 - 0_NC; AR20_020 - 0_NC; AR20_100 - 0_NC; AR20_200 - 0_NC; AR20_200 - AR20_020
4-(Trifuoromethyl)cinnamic acid	11.7	0.0009	3.0625	0.0072	AR20_100 - 0_NC; AR20_200 - 0_NC; AR20_200 - AR20_002; AR20_200 - AR20_020; AR20_200 - AR20_100
Lactic acid	9.1959	0.0022	2.6575	0.0138	0_NC - AR20_002; 0_NC - AR20_020; 0_NC - AR20_100; 0_NC - AR20_200; AR20_002 - AR20_100
Malic acid	9.1915	0.0022	2.6567	0.0138	AR20_002 - 0_NC; AR20_200 - 0_NC; AR20_002 - AR20_020; AR20_002 - AR20_100; AR20_200 - AR20_020; AR20_200 - AR20_100
Thymine	8.6268	0.0028	2.554	0.0155	AR20_002 - 0_NC; AR20_020 - 0_NC; AR20_100 - 0_NC; AR20_200 - 0_NC; AR20_200 - AR20_020
Guanine	7.1284	0.0055	2.2558	0.0277	AR20_002 - 0_NC; AR20_020 - 0_NC; AR20_100 - 0_NC; AR20_200 - 0_NC; AR20_100 - AR20_020
Leucine	6.8391	0.0064	2.1933	0.0291	0_NC - AR20_002; 0_NC - AR20_020; 0_NC - AR20_100; 0_NC - AR20_200
a-Tocopherol	6.3431	0.0083	2.0819	0.0345	AR20_002 - 0_NC; AR20_020 - 0_NC; AR20_100 - 0_NC; AR20_200 - 0_NC
Glutathione reduced	5.6293	0.0123	1.911	0.0472	AR20_002 - 0_NC; AR20_002 - AR20_020; AR20_002 - AR20_100; AR20_002 - AR20_200

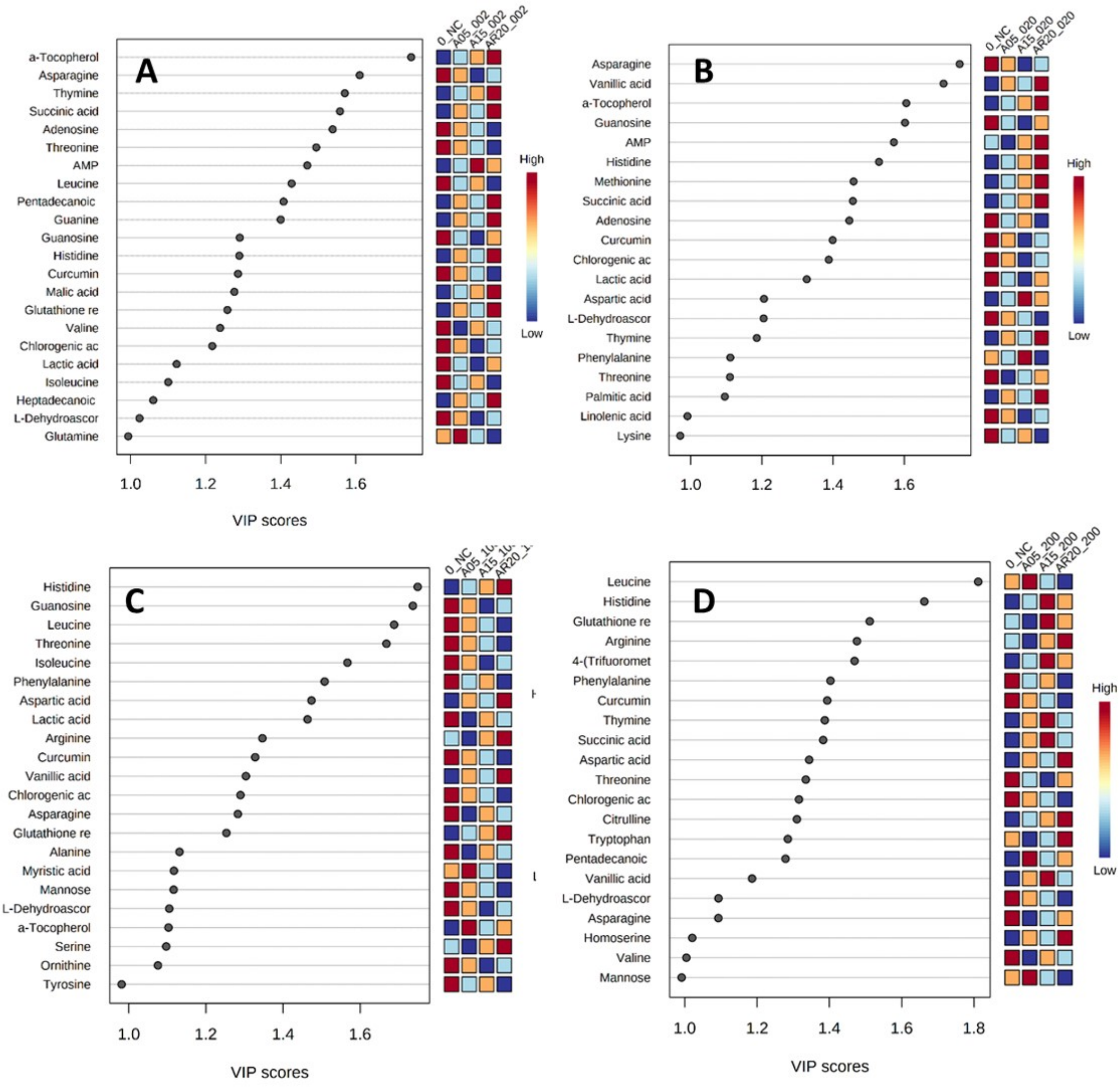

Figure S4. VIP scores from PLS-DA analysis of discriminating metabolites between unexposed controls and A5, A15 and AR20 treatments of different size at given concentration. The colored boxes on the right indicate the relative concentrations of the corresponding metabolite in each group under study. Treatments with: (A) 2 mg L^{-1} of $nTiO_2$ with size of 5nm (A5_002),15 nm (A15_002) and 20 nm (AR20_002); (B) 20 mg L^{-1} of $nTiO_2$ with size of 5nm (A5_020),15 nm (A15_020) and 20 nm (AR20_020); (C) 100 mg L^{-1} of $nTiO_2$ with size of 5nm (A5_100), 15 nm (A15_100) and 20 nm (AR20_100); (D) 200 mg L^{-1} of $nTiO_2$ with size of 5nm (A5_200),15 nm (A15_200) and 20 nm (AR20_200). Only metabolites with a VIP >1, regarded as significant are present.

Table S6. Important features identified by One-way ANOVA and Fisher's post-hoc analysis ($p < 0.05$) in *C. reinhardtii* exposed to 2 mg L^{-1} of $nTiO_2$ of different primary size: 5 nm (A5), 15 nm (A15) and 20 nm (AR20). Data were row-wise normalized using probabilistic quotient normalization by reference groups. non-transformed and autoscaled.

Metabolites	f.value	p.value	-log10(p)	FDR	Fisher's LSD
Guanosine	74.935	0	5.4706	0.0002	NC - A05_002; NC - A15_002; NC - AR20_002
Methionine	38.569	0	4.3776	0.001	A15_002 - A05_002; A05_002 - AR20_002; A05_002 - NC; A15_002 - AR20_002; A15_002 - NC; AR20_002 - NC
Uridine	31.245	0.0001	4.0405	0.0013	A15_002 - A05_002; A05_002 - AR20_002; A05_002 - NC; A15_002 - AR20_002; A15_002 - NC
Asparagine	29.854	0.0001	3.9684	0.0013	A05_002 - A15_002; A05_002 - AR20_002; NC - A05_002; NC - A15_002; NC - AR20_002
Leucine	24.839	0.0002	3.6803	0.0021	A15_002 - A05_002; NC - A05_002; A15_002 - AR20_002; NC - A15_002; NC - AR20_002
a-Tocopherol	22.986	0.0003	3.5604	0.0023	AR20_002 - A05_002; A05_002 - NC; AR20_002 - A15_002; A15_002 - NC; AR20_002 - NC
Glutathione reduced	14.887	0.0012	2.9107	0.0084	A05_002 - A15_002; A05_002 - NC; AR20_002 - A15_002; AR20_002 - NC
Valine	14.466	0.0014	2.8692	0.0084	NC - A05_002; NC - A15_002; NC - AR20_002
Lactic acid	13.254	0.0018	2.7442	0.01	NC - A05_002; NC - A15_002; NC - AR20_002
Guanine	12.5	0.0022	2.6615	0.0109	AR20_002 - A05_002; AR20_002 - A15_002; AR20_002 - NC
AMP	11.292	0.003	2.5204	0.0137	A05_002 - NC; A15_002 - NC; AR20_002 - NC
Arginine	10.064	0.0043	2.3644	0.018	A05_002 - A15_002; A05_002 - NC; AR20_002 - A15_002; AR20_002 - NC
Succinic acid	9.5826	0.005	2.2991	0.0193	AR20_002 - A05_002; A05_002 - NC; AR20_002 - A15_002; A15_002 - NC; AR20_002 - NC
Thymine	9.2842	0.0055	2.2574	0.0195	AR20_002 - A05_002; A05_002 - NC; A15_002 - NC; AR20_002 - NC
Threonine	9.1185	0.0058	2.2338	0.0195	A05_002 - A15_002; A05_002 - AR20_002; NC - A15_002; NC - AR20_002
Benzoic acid	8.7315	0.0066	2.1775	0.0208	A05_002 - A15_002; A05_002 - AR20_002; A05_002 - NC
Adenosine	8.4455	0.0073	2.1346	0.0216	NC - A05_002; NC - A15_002; NC - AR20_002
4-Trifuoromethylcinnamic acid	7.7567	0.0094	2.0269	0.0261	A05_002 - NC; A15_002 - NC; AR20_002 - NC

Table S7. Important features identified by One-way ANOVA and Fisher's post-hoc analysis ($p < 0.05$) in *C. reinhardtii* exposed to 20 mg L^{-1} of $nTiO_2$ of different primary size: 5 nm (A5), 15 nm (A15) and 20 nm (AR20). Data were row-wise normalized using probabilistic quotient normalization by reference groups. non-transformed and autoscaled.

Metabolites	f.value	p.value	-log10(p)	FDR	Fisher's LSD
Guanosine	35.03	0.0001	4.2227	0.003	NC - A05_020; NC - A15_020; NC - AR20_020
Phenylalanine	13.67	0.0016	2.7876	0.0352	A15_020 - A05_020; NC - A05_020; A15_020 - AR20_020; NC - AR20_020
Ornithine	10.8	0.0035	2.459	0.0352	A15_020 - A05_020; NC - A05_020; A15_020 - AR20_020; NC - AR20_020
Arachidic acid	10.72	0.0036	2.4496	0.0352	A05_020 - AR20_020; A05_020 - NC; A15_020 - NC; AR20_020 - NC
Vanillic acid	10.06	0.0043	2.3635	0.0352	A05_020 - NC; A15_020 - NC; AR20_020 - NC
Asparagine	10	0.0044	2.3559	0.0352	A05_020 - A15_020; NC - A15_020; NC - AR20_020
AMP	9.387	0.0053	2.2719	0.0352	A15_020 - A05_020; AR20_020 - A05_020; A15_020 - NC; AR20_020 - NC
Lactic acid	9.23	0.0056	2.2498	0.0352	NC - A05_020; NC - A15_020; NC - AR20_020
Valine	8.371	0.0075	2.1233	0.0418	AR20_020 - A05_020; NC - A05_020; NC - A15_020

Table S8. Important features identified by One-way ANOVA and Fisher's post-hoc analysis ($p < 0.05$) in *C. reinhardtii* exposed to 100 mg L^{-1} of $nTiO_2$ of different primary size: 5 nm (A5), 15 nm (A15) and 20 nm (AR20). Data were row-wise normalized using probabilistic quotient normalization by reference groups. non-transformed and autoscaled.

Metabolites	f.value	p.value	-log10(p)	FDR	Fisher's LSD
Valine	77.458	0	5.526	0.0001	A15_100 - A05_100; AR20_100 - A05_100; NC - A05_100; AR20_100 - A15_100; NC - A15_100; NC - AR20_100
Lactic acid	74.12	0	5.4523	0.0001	NC - A05_100; NC - A15_100; NC - AR20_100
Lysine	49.269	0	4.776	0.0003	A15_100 - A05_100; AR20_100 - A05_100; NC - A05_100; NC - A15_100; NC - AR20_100
Leucine	44.197	0	4.5985	0.0003	NC - A05_100; NC - A15_100; NC - AR20_100
Guanine	39.986	0	4.436	0.0003	A05_100 - A15_100; A05_100 - AR20_100; A05_100 - NC; A15_100 - NC; AR20_100 - NC
Guanosine	38.905	0	4.3917	0.0003	A05_100 - A15_100; A05_100 - AR20_100; NC - A05_100; NC - A15_100; NC - AR20_100
Tryptophan	37.183	0	4.3187	0.0003	A05_100 - A15_100; AR20_100 - A05_100; AR20_100 - A15_100; NC - A15_100; AR20_100 - NC
Benzoic acid	32.192	0.0001	4.088	0.0005	A05_100 - A15_100; A05_100 - AR20_100; A05_100 - NC
4-Trifuoromethylcinnamic acid	25.124	0.0002	3.698	0.0011	A05_100 - A15_100; A05_100 - AR20_100; A05_100 - NC; A15_100 - NC; AR20_100 - NC
Methionine	24.581	0.0002	3.6641	0.0011	A05_100 - AR20_100; A05_100 - NC; A15_100 - AR20_100; A15_100 - NC; AR20_100 - NC
Alanine	23.629	0.0002	3.603	0.0011	A15_100 - A05_100; NC - A05_100; NC - A15_100; NC - AR20_100
Thymine	20.75	0.0004	3.4039	0.0015	A05_100 - A15_100; A05_100 - AR20_100; A05_100 - NC; A15_100 - NC; AR20_100 - NC
Succinic acid	20.483	0.0004	3.3842	0.0015	A05_100 - A15_100; A05_100 - AR20_100; A05_100 - NC; A15_100 - NC; AR20_100 - NC
Arachidic acid	20.372	0.0004	3.376	0.0015	A05_100 - A15_100; A05_100 - AR20_100; A05_100 - NC; A15_100 - NC; AR20_100 - NC
Asparagine	19.072	0.0005	3.2764	0.0018	NC - A05_100; NC - A15_100; NC - AR20_100

Threonine	14.557	0.0013	2.8782	0.0041	NC - A05_100; NC - A15_100; NC - AR20_100
Glutamic acid	10.305	0.004	2.3961	0.0118	AR20_100 - A05_100; NC - A05_100; AR20_100 - A15_100; NC - A15_100
Phenylalanine	9.2832	0.0055	2.2573	0.0154	NC - A05_100; A15_100 - AR20_100; NC - AR20_100
Histidine	8.7963	0.0065	2.1871	0.0171	AR20_100 - A05_100; A15_100 - NC; AR20_100 - NC
Citrulline	8.5916	0.007	2.1567	0.0174	A05_100 - A15_100; A05_100 - NC; AR20_100 - NC
Isoleucine	7.858	0.0091	2.0432	0.0208	NC - A05_100; NC - A15_100; NC - AR20_100
Stearic acid	7.8244	0.0092	2.0378	0.0208	A05_100 - AR20_100; A05_100 - NC; A15_100 - NC
a-Tocopherol	7.03	0.0124	1.9056	0.027	A05_100 - NC; AR20_100 - NC
AMP	6.625	0.0146	1.8342	0.0305	A15_100 - A05_100; AR20_100 - A05_100; NC - A05_100
Aspartic acid	6.1109	0.0182	1.7392	0.0353	A05_100 - NC; AR20_100 - NC
Vanillic acid	6.0758	0.0185	1.7325	0.0353	A05_100 - NC; AR20_100 - NC
Myristic acid	5.9549	0.0195	1.7093	0.0353	A05_100 - A15_100; A05_100 - AR20_100
Malic acid	5.9295	0.0198	1.7044	0.0353	A05_100 - A15_100; A05_100 - AR20_100; A05_100 - NC
Proline	5.6025	0.0229	1.6399	0.0395	AR20_100 - A05_100; NC - A05_100
Tyrosine	5.0301	0.0301	1.5211	0.0486	NC - A05_100; A15_100 - AR20_100; NC - AR20_100
Curcumin	5.0293	0.0301	1.5209	0.0486	NC - A05_100; NC - A15_100; NC - AR20_100

Table S9. Important features identified by One-way ANOVA and Fisher's post-hoc analysis ($p < 0.05$) in *C. reinhardtii* exposed to 200 mg L^{-1} of $nTiO_2$ of different primary size: 5 nm (A5), 15 nm (A15) and 20 nm (AR20) Data were row-wise normalized using probabilistic quotient normalization by reference groups. non-transformed and autoscaled.

Metabolites	f.value	p.value	-log10(p)	FDR	Fisher's LSD
AMP	115.4	0	6.1971	0	200A15 - 200A5; 200AR20 - 200A15; NC - 200A15; 200AR20 - 200A5; NC - 200A5
Lysine	89.34	0	5.7653	0	200A15 - 200A5; 200A15 - 200AR20; 200AR20 - 200A5; NC - 200A5; NC - 200AR20
Lactic acid	51.92	0	4.8621	0	200AR20 - 200A15; NC - 200A15; 200AR20 - 200A5; NC - 200A5; NC - 200AR20
Arginine	34.13	0.0001	4.1813	0	200A15 - 200A5; 200A15 - NC; 200AR20 - 200A5; NC - 200A5; 200AR20 - NC
Thymine	31.62	0.0001	4.0595	0	200A15 - NC; 200A5 - NC; 200AR20 - NC
Succinic acid	24.18	0.0002	3.6384	0	200A15 - NC; 200A5 - NC; 200AR20 - NC
Asparagine	23.47	0.0003	3.5925	0	NC - 200A15; 200AR20 - 200A5; NC - 200A5; NC - 200AR20
Leucine	22.06	0.0003	3.4976	0	200A5 - 200A15; NC - 200A15; 200A5 - 200AR20; NC - 200AR20
Methionine	20.38	0.0004	3.3766	0	200A15 - 200AR20; 200A15 - NC; 200A5 - 200AR20; 200A5 - NC; 200AR20 - NC
Tryptophan	20.32	0.0004	3.3724	0	200A15 - 200A5; 200AR20 - 200A15; 200AR20 - 200A5; NC - 200A5; 200AR20 - NC
Histidine	20.13	0.0004	3.3579	0	200A15 - 200A5; 200A15 - NC; 200AR20 - 200A5; 200AR20 - NC
Threonine	16.46	0.0009	3.0572	0	NC - 200A15; NC - 200A5; NC - 200AR20
Glutathione reduced	14.92	0.0012	2.9141	0	200A15 - 200A5; 200A15 - NC; 200AR20 - 200A5; 200AR20 - NC
Alanine	13.2	0.0018	2.7386	0.01	200A15 - 200A5; 200AR20 - 200A5; NC - 200A5
4-(Trifuoromethyl)cinnamic acid	13.09	0.0019	2.7265	0.01	200A15 - 200A5; 200A15 - NC; 200A5 - NC; 200AR20 - NC

Myristic acid	12.06	0.0024	2.6112	0.01	200A5 - 200A15; 200AR20 - 200A15; NC - 200A15; 200A5 - 200AR20
Valine	10.78	0.0035	2.4566	0.01	200A15 - 200A5; NC - 200A5; NC - 200AR20
Pentadecanoic acid	9.018	0.006	2.2193	0.02	200A15 - NC; 200A5 - NC; 200AR20 - NC
Proline	8.602	0.0069	2.1581	0.02	200A15 - 200A5; 200A15 - NC; 200AR20 - 200A5; NC - 200A5
Vanillic acid	7.396	0.0108	1.9678	0.03	200A15 - NC; 200A5 - NC; 200AR20 - NC
Malic acid	6.514	0.0153	1.8142	0.04	200A5 - 200A15; 200AR20 - 200A15; 200A5 - NC; 200AR20 - NC

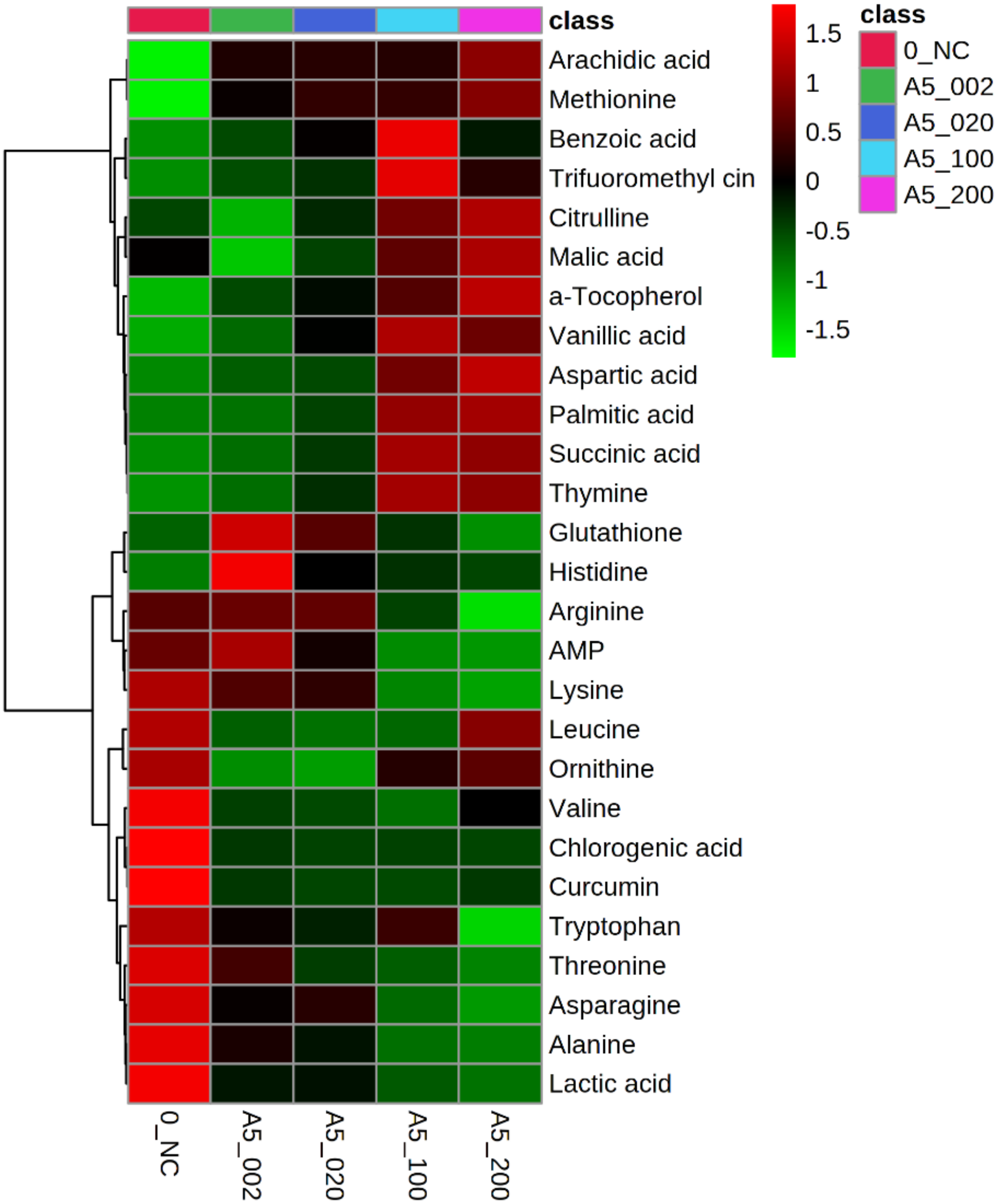

Figure S5. Heat map presentation of the significantly altered metabolites (ANOVA and PLS-DA) *in C. reinhardtii* treated with increasing concentrations of $nTiO_2$ with primary size of 5 nm. Clustering by Euclidean distance and Ward clustering algorithm. Treatments: $nTiO_2$ 2 mg L^{-1} (A5_2), 20 mg L^{-1} (A5_20), 100 mg L^{-1} (A5_100) and 200 mg L^{-1} (A5_200).

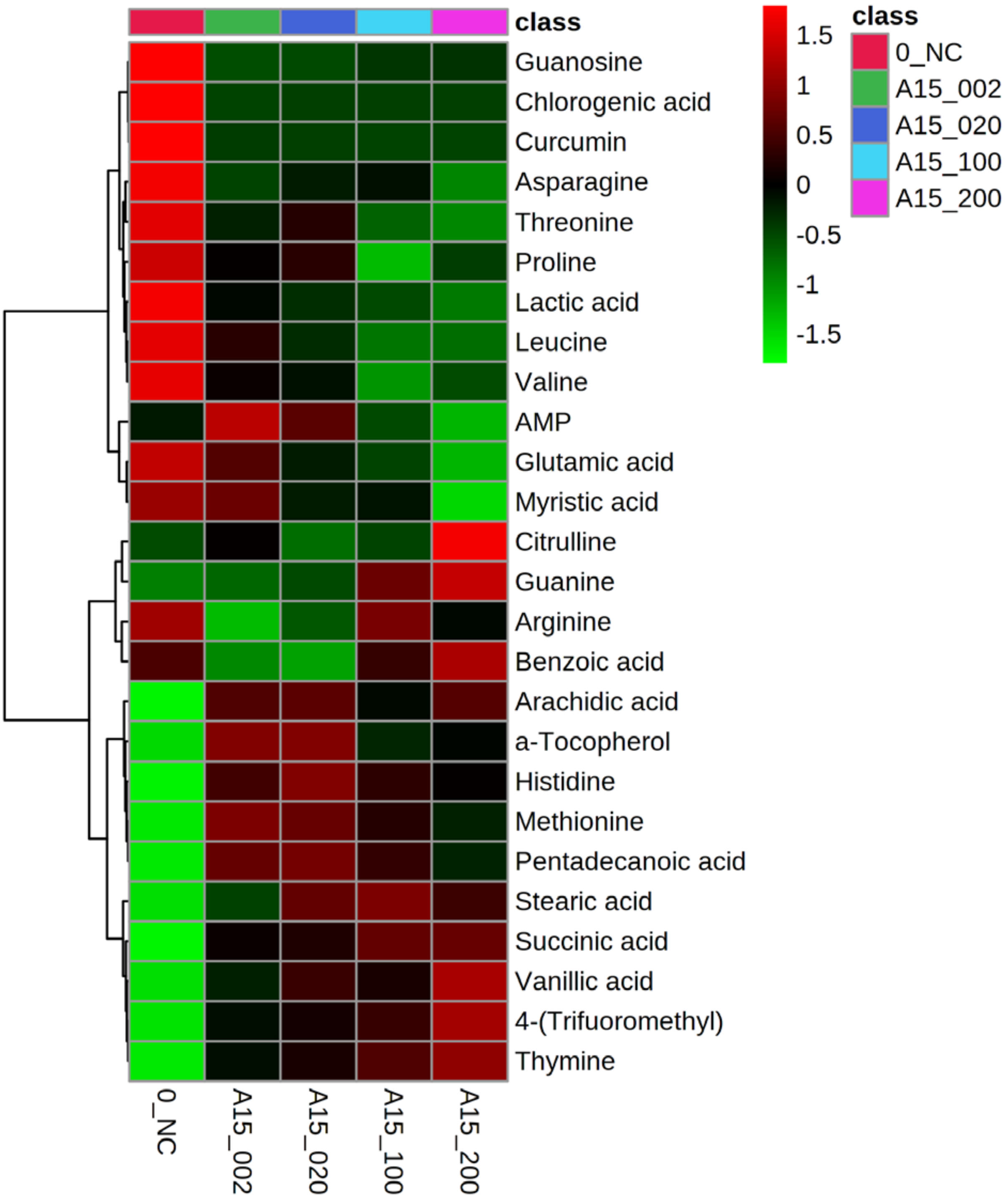

Figure S6. Heat map presentation of the altered responsive metabolites in *C. reinhardtii* treated with increasing concentrations of $nTiO_2$ with primary size of 15 nm. Clustering by Euclidean distance and Ward clustering algorithm. Treatments: $nTiO_2$ 2 mg L^{-1} (A15_2), 20 mg L^{-1} (A15_20), 100 mg L^{-1} (A15_100) and 200 mg L^{-1} (A15_200).

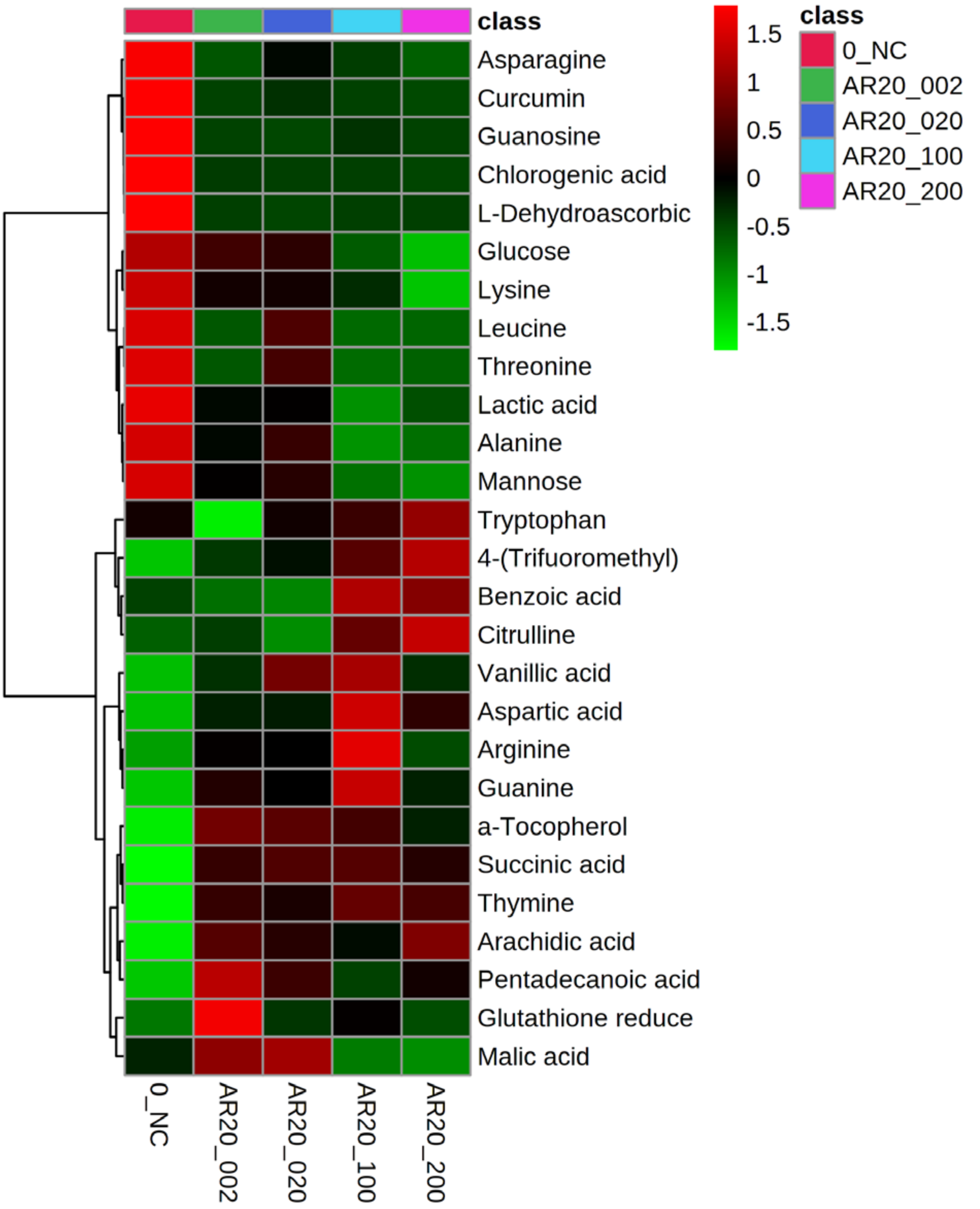

Figure S7. Heat map presentation of the altered metabolites in *C. reinhardtii* treated with increasing concentrations of $nTiO_2$ with primary size of 20 nm. Clustering by Euclidean distance and Ward clustering algorithm. Treatments: $nTiO_2$ 2 mg L^{-1} (AR20_2), 20 mg L^{-1} (AR20_20), 100 mg L^{-1} (AR20_100) and 200 mg L^{-1} (AR20_200).

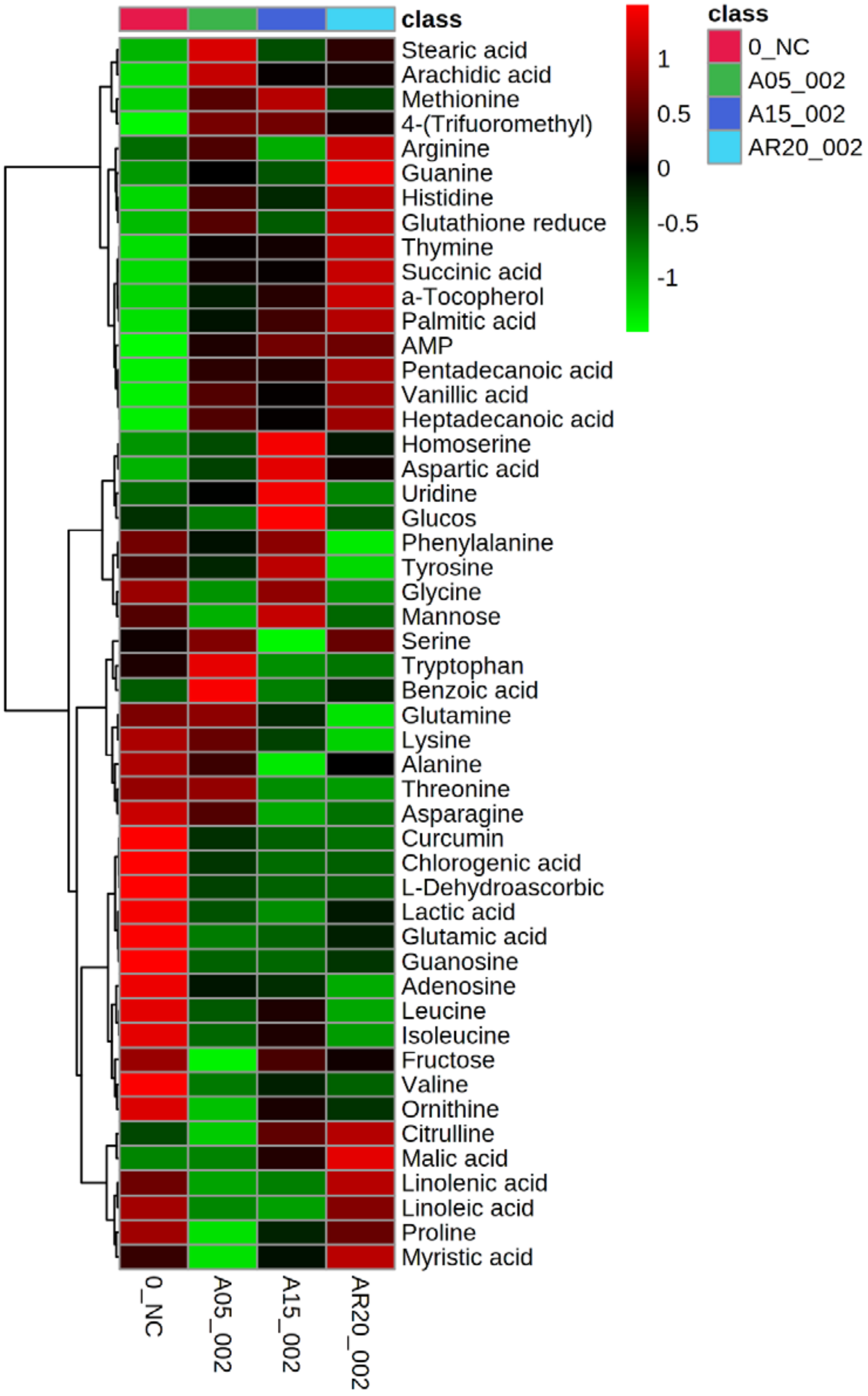

Figure S8. Heat map presentation of the altered metabolites in *C. reinhardtii* treated with $nTiO_2$ at different primary sizes. Clustering by Euclidean distance and Ward clustering algorithm. Treatments: $nTiO_2$ 2 mg L^{-1} with size of 5nm (A5_002), 15 nm (A15_002) and 20 nm (AR20_002).

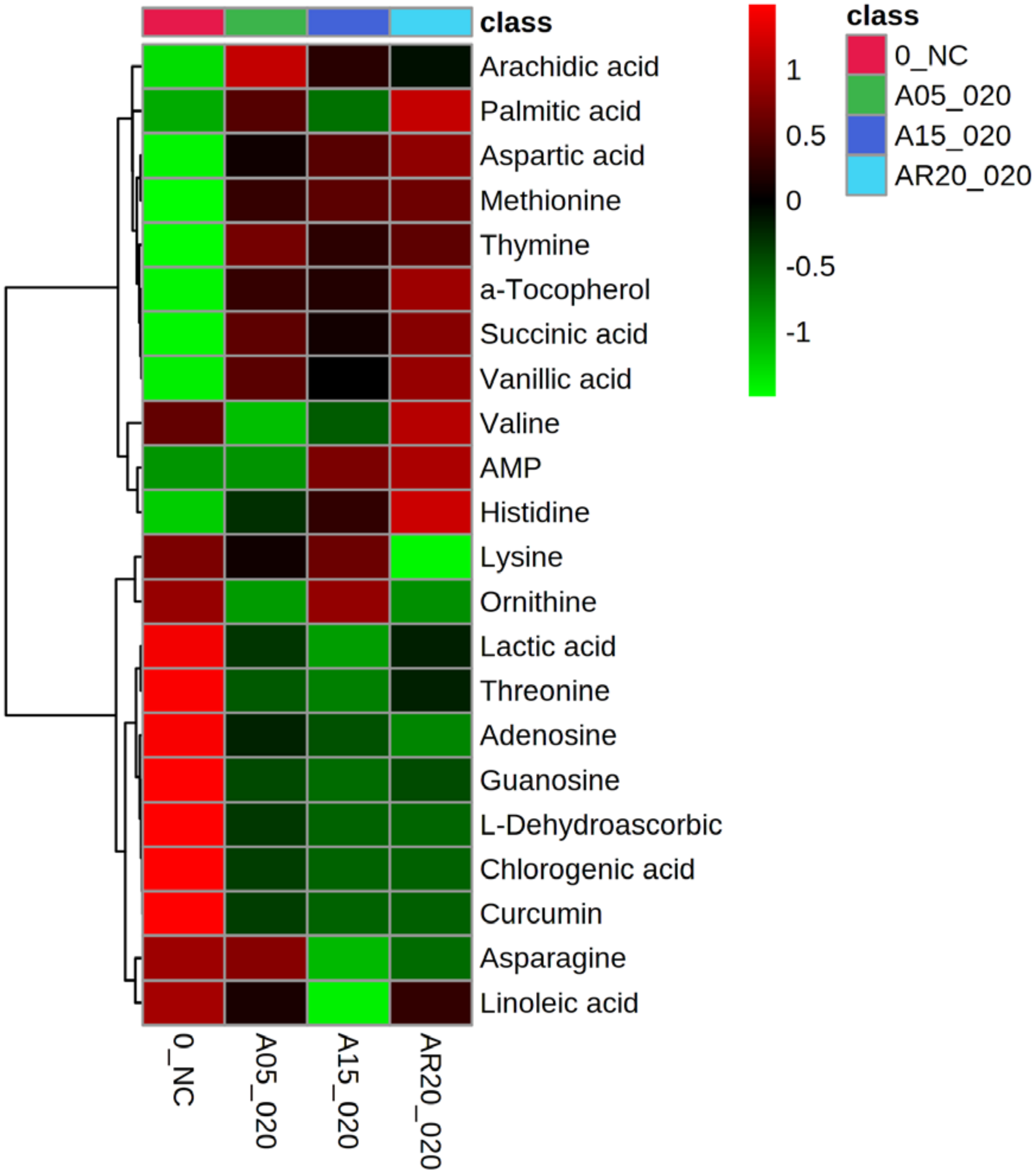

Figure S9. Heat map presentation of the altered metabolites in *C. reinhardtii* treated with $nTiO_2$ at different primary sizes. Clustering by Euclidean distance and Ward clustering algorithm. Treatments: $nTiO_2$ 20 mg L^{-1} with size of 5nm (A5_020), 15 nm (A15_020) and 20 nm (AR20_020).

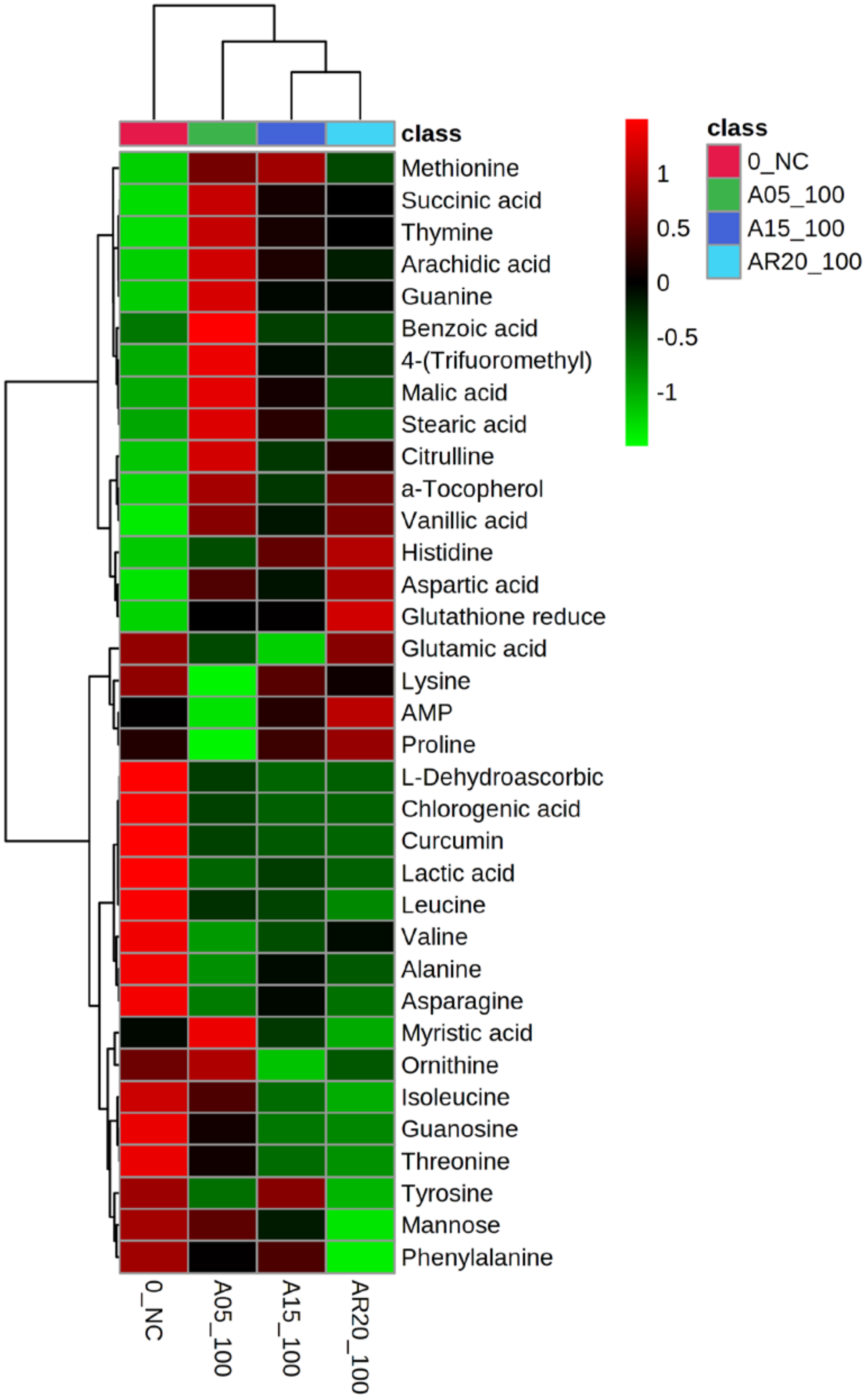

Figure S10. Heat map presentation of the altered metabolites in *C. reinhardtii* treated with $nTiO_2$ at different primary sizes. Clustering by Euclidean distance and Ward clustering algorithm. Treatments: $nTiO_2$ 100 mg L^{-1} with size of 5nm (A5_100), 15 nm (A15_100) and 20 nm (AR20_100).

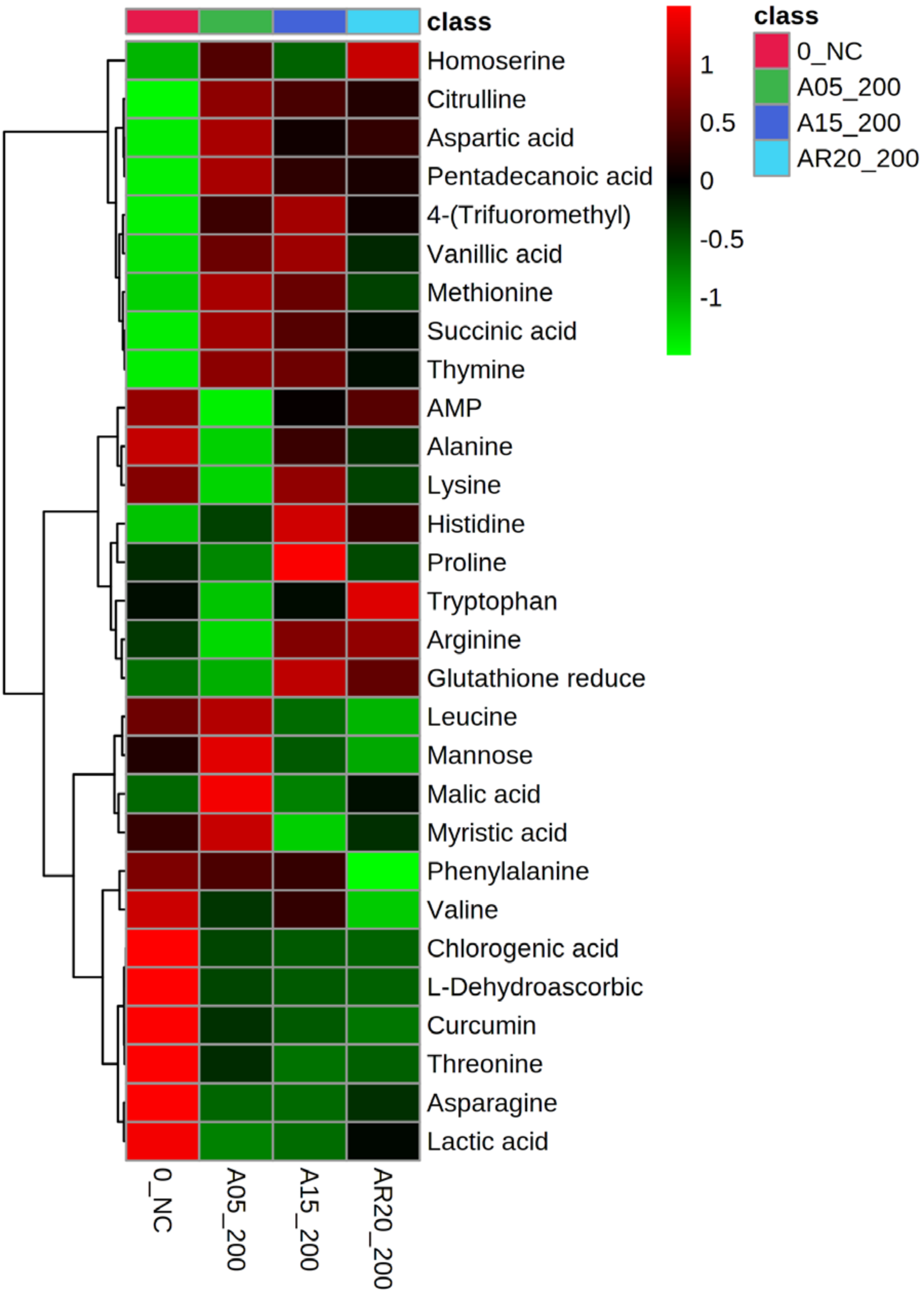

Figure S11. Heat map presentation of the altered metabolites in *C. reinhardtii* treated with $nTiO_2$ at different primary sizes. Clustering by Euclidean distance and Ward clustering algorithm. Treatments: $nTiO_2$ 200 mg L^{-1} with size of 5nm (A5_200), 15 nm (A15_200) and 20 nm (AR20_200).

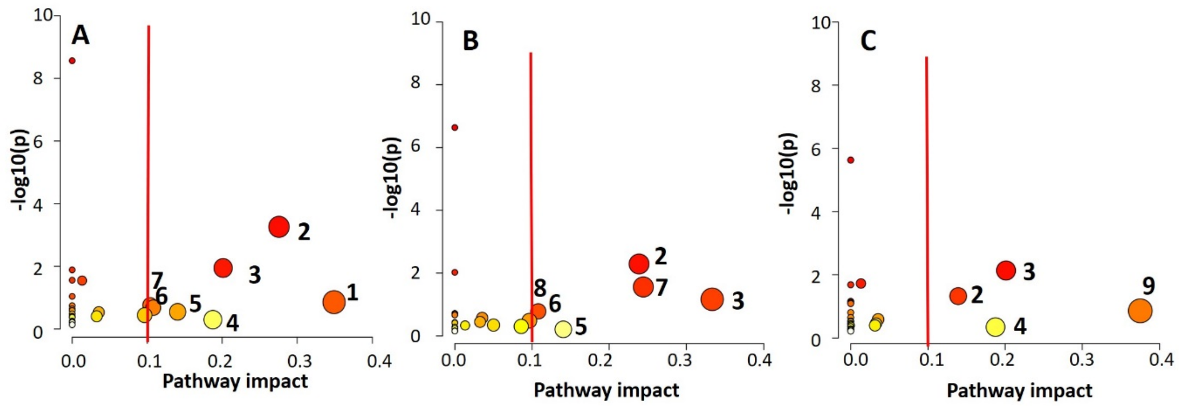

Figure S12. Pathway analysis for metabolites with altered abundance in *C. reinhardtii* exposed to increasing concentrations of (A) A5, (B) A15 and (C) AR20. The node color is based on its p-value and changes from red to yellow with the increase of p-value. The node size reflects the pathway impact values, with bigger nodes corresponding to high impact values. Affected pathways: 1: Glutathione metabolism; 2: Arginine biosynthesis; 3: Alanine, aspartate and glutamate metabolism; 4: Tryptophan metabolism; 5: Cysteine and methionine metabolism; 6: Purine metabolism; 7: Arginine and proline metabolism, 8: Histidine metabolism and 9: Phenylalanine metabolism. The responsive metabolites obtained in algal treatments with different concentrations were used for the pathway analysis.

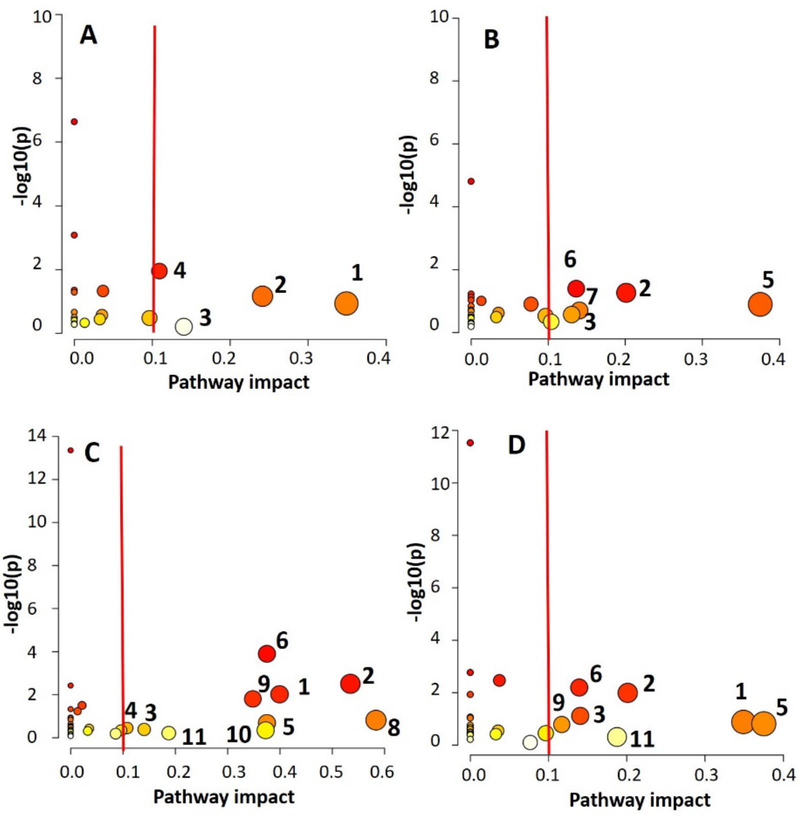

Figure S13. Pathway analysis for metabolites with altered abundance in *C. reinhardtii* exposed to different concentrations of $nTiO_2$ with size of 5, 15 and 20 nm (A) 2 mgL^{-1}, (B) 20 mgL^{-1}, (C) 100 $mgL^{-1}$and (C) 200 mgL^{-1} $nTiO_2$. The node color is based on its p-value and changes from red to yellow with the increase of p-value. The node size reflects the pathway impact values, with bigger nodes corresponding to high impact values. Affected pathways: 1: Glutathione metabolism, 2: Alanine, aspartate and glutamate metabolism; 3: Cysteine and methionine metabolism; 4: Purine metabolism; 5: Phenylalanine metabolism; 6: Arginine biosynthesis; 7. alpha-Linolenic acid metabolism; 8 Isoquinoline alkaloid biosynthesis; 9: Arginine and proline metabolism; 10: Tyrosine metabolism and 11. Tryptophan metabolism. The responsive metabolites obtained in algal treatments with different primary size were used for the pathway analysis.

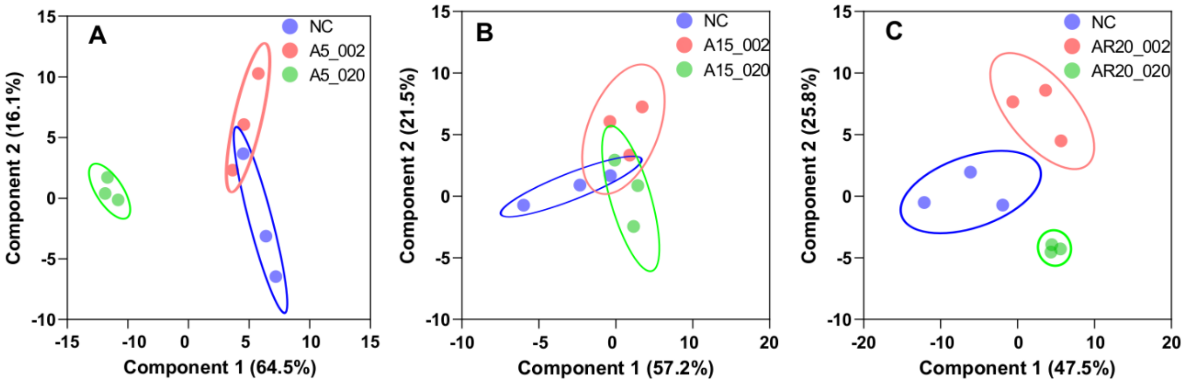

Figure S14. PCA analysis of transcript expression of *C. reinhardtii* exposed to 2 and 20 mg L^{-1} of three types of $nTiO_2$. (A) Treatments with A5 at 2 mg L^{-1} (A5_002) and 20 mg L^{-1} (A5_020). (B) Treatments with A15 at 2 mg L^{-1} (A5_002) and 20 mg L^{-1} (A5_020). (C) Treatments with AR20 at 2 mg L^{-1} (A5_002) and 20 mg L^{-1} (A5_020); Negative control (NC).

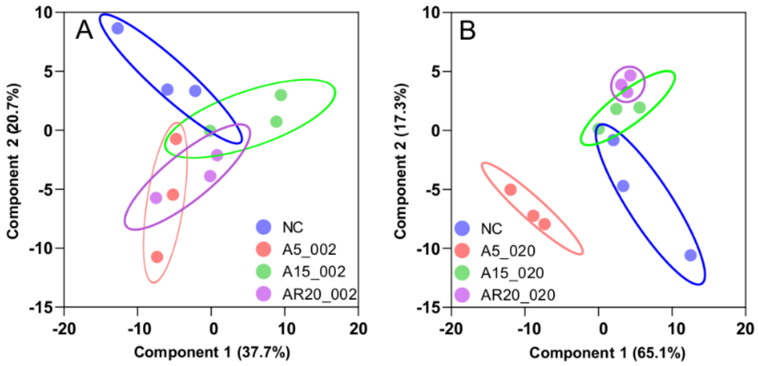

Figure S15. PCA analysis of transcript expression of *C. reinhardtii* exposed to $nTiO_2$ of 5, 15 and 20 nm primary size at 2 and 20 mg L^{-1}. (A) Treatments with A5 at 2 mg L^{-1} of A5 (A5_002), A15 (A 15_002) and AR20 (AR20_002). (B) Treatments with A5 at 20 mg L^{-1} of A5 (A5_020), A15 (A 15_020) and AR20 (AR20_020); Negative control (NC).

Table S10. Number of significantly dysregulated genes in metabolic pathways (MapMan) in *C. reinhardtii* after 72 h exposure to A5, A15 and AR20 at 2 and 20 mgL^{-1}.

Metabolic Pathways	Number of genes					
	A5		A15		AR20	
	2 mgL^{-1}	20 mgL^{-1}	2 mgL^{-1}	20 mgL^{-1}	2 mgL^{-1}	20 mgL^{-1}
Amino acid metabolism	1	4	0	0	0	0
Carbohydrates	0	9	0	0	1	2
Metal binding	0	1	0	0	0	1
Photosynthesis	1	2	0	0	0	2
Stress	0	9	0	0	1	0
Transport	1	11	4	4	1	3
Energy metabolism	0	1	1	0	0	0
Cell processes	0	2	0	0	0	0
Other metabolisms	0	9	3	5	0	0

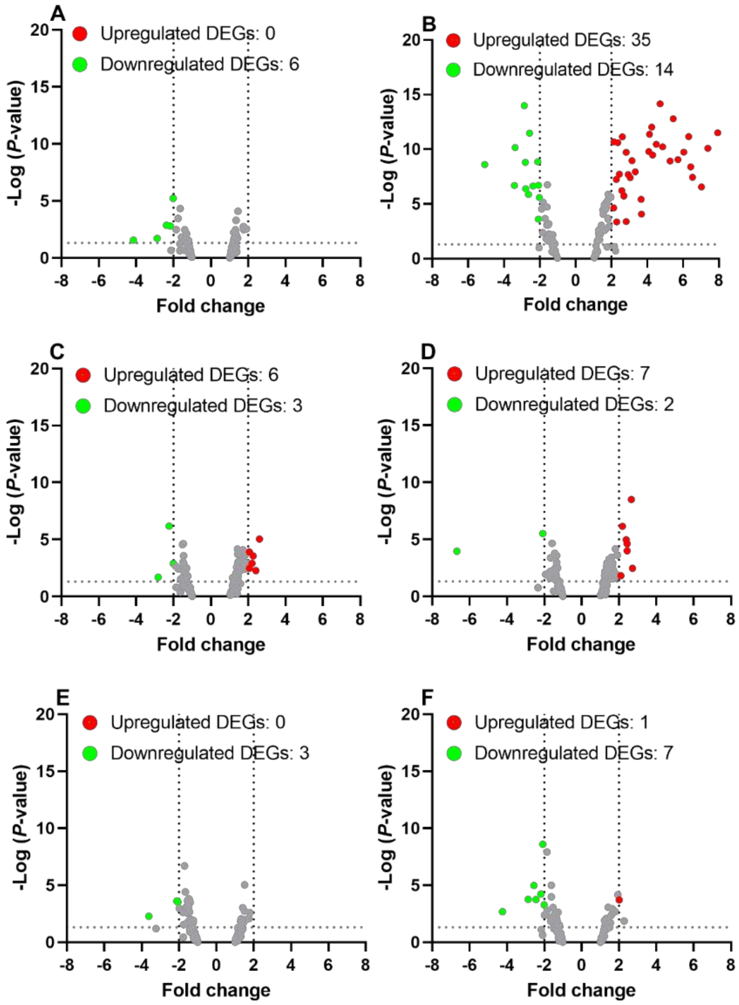

Figure S16. Volcano plot of differentially expressed genes (DGEs) of *C. reinhardtii* after exposure to three $nTiO_2$ at 2 and 20 mg L^{-1} based on a significance threshold under FDR <0.05 associated with fold change value of 2 or more. The number of upregulated (depicted in red dots) and downregulated (depicted in green dot) transcripts in *C. reinhardtii* after exposure to A5 at 2 mg L^{-1} (A) and 20 mg L^{-1} (B); A15 at 2 mg L^{-1} (C) and 20 mg L^{-1} (D); AR20 at 2 mg L^{-1} (E) and 20 mg L^{-1} (F) respectively. Each point represents the average value of one transcript in three replicate experiments. The expression difference is considered significant for a Log2 fold change value ≥ 2 (gray broken vertical lines) and for a *p* value of 0.05 [log(FDR) of 1.3, gray broken horizontal line]. Transcripts that were not found to be significantly altered are presented as grey dots.

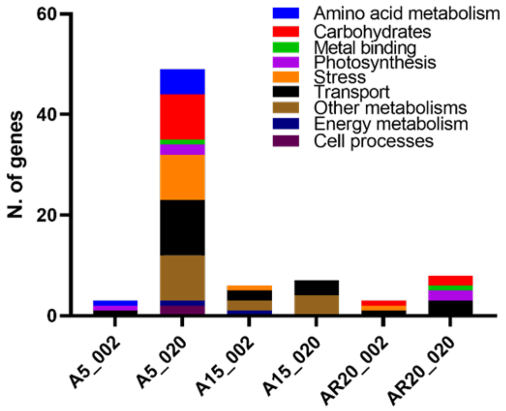

Figure S17. Distribution of number of significantly dysregulated transcripts corresponding to pathways in *C. reinhardtii* exposed to A5, A15 and AR20 at 2 mg L^{-1}, and 20 mg L^{-1}.

At higher exposure concentration of 20 mgL^{-1} of A5, the expression level of the transcripts involved in transport (11), stress (9), carbohydrate (9) and amino acid metabolisms (5) were strongly responding. Transcripts coding for ‘cell processes’ and ‘photosynthesis’ showed the lowest importance. Despite that exposure to A5 dysregulated a larger number of genes than A15 and AR20 which exhibited the similar categories of dysregulated genes.
